# Optimized RT-qPCR and a novel normalization method for validating circulating miRNA biomarkers in ageing-related diseases

**DOI:** 10.1038/s41598-023-47971-3

**Published:** 2023-11-27

**Authors:** Andrew Want, Karolina Staniak, Wioleta Grabowska-Pyrzewicz, Aleksandra Fesiuk, Anna Barczak, Tomasz Gabryelewicz, Agnieszka Kulczyńska-Przybik, Barbara Mroczko, Urszula Wojda

**Affiliations:** 1 Laboratory of Preclinical Testing of Higher Standard, Nencki Institute of Experimental Biology, Polish Academy of Sciences, Warsaw, Poland; 2 Dementia Diseases Unit, Mossakowski Medical Research Institute, Polish Academy of Sciences, Warsaw, Poland; 3 Department of Neurodegeneration Diagnostics, Faculty of Medicine, Medical University of Bialystok, Białystok, Poland

**Keywords:** Biochemistry, Biological techniques, Biotechnology, Computational biology and bioinformatics, Neuroscience, Biomarkers, Diseases, Molecular medicine

## Abstract

Circulating miRNAs have potential as minimally invasive biomarkers for diagnosing various diseases, including ageing-related disorders such as Alzheimer’s disease (AD). However, the lack of standardization in the common analysis method, RT-qPCR, and specifically in the normalization step, has resulted in inconsistent data across studies, hindering miRNA clinical implementation as well as basic research. To address this issue, this study proposes an optimized protocol for key steps in miRNA profiling, which incorporates absorbance-based haemolysis detection for assessing sample quality, double spike-in controls for miRNA isolation and reverse transcription, and the use of 7 stable normalizers verified in an aging population, including healthy subjects and individuals at different stages of Alzheimer's disease (140 subjects). The stability of these 7 normalizers was demonstrated using our novel method called BestmiRNorm for identifying optimal normalizers. BestmiRNorm, developed utilizing the Python programming language, enables the assessment of up to 11 potential normalizers. The standardized application of this optimized RT-qPCR protocol and the recommended normalizers are crucial for the development of miRNAs as biomarkers for AD and other ageing-related diseases in clinical diagnostics and basic research.

## Introduction

The identification of biomarkers circulating in blood that can be detected using cost-effective and minimally invasive procedures is expected to become a breakthrough innovation for improving the diagnostics and prognostics of many diseases, including ageing-related disorders such as Alzheimer’s disease (AD). The development of AD diagnostics is a particularly good example of such efforts. Currently, AD diagnostics are based on the assessment of dementia symptoms supported by the AD-associated biomarkers amyloid Aβ and tau and phosphorylated tau (p-tau/tau), whose levels are measured in cerebrospinal fluid (CSF) with immunoassays and imaging methods in the brains of living patients^1,2^. The invasiveness of lumbar puncture for CSF assays, unsuitability for repeated measures, and high costs of brain imaging significantly limit the broad applicability of these assays, driving the search for robust, easily accessible blood-based biomarkers. Specifically, AD and other dementias are characterized by protracted latent periods in which diagnosis is a significant challenge. Because early AD detection is the key to more effective therapy, blood-based diagnostic biomarkers are critically needed for early symptomatic AD stages, as well as prognostic biomarkers that could detect the state of predementia, evaluate AD risk and allow for prophylaxis. Broadly accessible, low-intervention biomarkers are also necessary for recruiting early AD patients for clinical trials of new therapies and repeated assessments of response to treatment.

To meet these needs, considerable research effort has been invested in identifying brain-derived AD-associated molecules such as Aβ and p-tau/tau in blood^3,4^. However, such molecules are present in low concentrations in blood, and moreover, their levels can be altered by molecules released from peripheral cells, making detection difficult despite application of highly sensitive assays.

New biomarker paradigms for blood plasma have also been investigated recently. Among several approaches tested, in the last decade, many reports have confirmed the biomarker capacity of circulating microRNAs (miRNAs) for AD as well as for other diseases^5^. miRNAs are short, noncoding, ubiquitous translational regulators implicated in a vast array of cellular processes. In humans, over 2500 miRNAs epigenetically regulate almost 60% of transcripts by RNA interference, eventually causing repression and degradation of target RNA^6^. miRNAs are released from cells in membranous extracellular vesicles or protected from degradation in protein or lipid complexes and can act as messengers in intercellular signalling^7–10^. An increasing number of reports have shown the presence of miRNAs in biofluids (plasma, serum, saliva, urine, tear, cerebrospinal fluid), their distinct composition in different fluid types and alteration of miRNA patterns between healthy subjects and individuals with different physiopathological conditions^11,12^. Given miRNA stable abundance in circulation, ability to cross the blood–brain barrier, mediating brain-blood cross-talk^13,14^ and availability of well-established technical methods for their isolation and measurements, miRNAs seem particularly promising as circulating biomarkers for AD and other diseases. A number of potential biomarker candidates have been proposed, but the studies lack consistency, mainly because of a lack of methodological standardization in miRNA analysis^5^.

In particular, after initial high-throughput screening and identification of candidate circulating miRNAs, the most sensitive method of choice for verification of the selected candidates is reverse transcription-quantitative polymerase chain reaction (RT-qPCR). Analysis of miRNA by RT-qPCR is a multistep procedure that includes blood plasma sample acquisition, sample quality checking, miRNA isolation, reverse transcription, qPCR, and data analysis. Each of these steps involves its own potential for confounding and requires proper quality controls. Currently, a variety of methods and controls are employed^15^. Furthermore, for proper interpretation of RT-qPCR data, normalisation with appropriate controls is critical. This is also compounded by the variation in normalisation methods (ΔC_q_ and ΔΔC_q_ being two of the most common), where it is apparent that the choice of normalisation method and normalisers can drastically alter the results^16^.

When measuring DNA/RNA in noncellular tissue, such as blood, standard normalisers such as glyceraldehyde-3-phosphate dehydrogenase (GAPDH) or β-actin are not physiologically relevant. This has resulted in the use of a collection of methods for identifying optimal normalisers and a patchwork of applied normalisers across the literature. The use of endogenous miRNAs as normalisers is one such method because their expression is considered to be affected by the same variables as the expression of target miRNAs. For optimal normalisation, RNA molecules that are considered to be stable under the experimental conditions should be employed. High-throughput technologies (such as microarrays) are typically normalized using a global mean of miRNA C_q_ values, and this is considered to be robust where large numbers of miRNAs are being analyzed. However, in smaller-scale experiments, such as using RT-qPCR, the grand mean is less reliable, so optimal normalisers must be selected from a broader panel within the scope of the experiment. For extracellular miRNAs, this often results in each study determining the optimal normalisers within the context of their own experiments. Moreover, three major algorithms are presently available for the determination of appropriate normalizers: NormFinder, GeNorm and Bestkeeper^17–19^, which can identify normalisers with the lowest variance or the optimal number of normalisers using model-based or pairwise comparison methods. Recently, a method was also described for combining these approaches into a composite measure^20^. All of the current, widely used methods have the same limitation of computational inefficiency as the number of normalisers being assessed increases.

Here, we examine key features of common analytical pipelines for miRNA in blood plasma using RT-qPCR and highlight areas where current standard practice needs optimization. These include recommendations for measurement of sample haemolysis and use of paired exogenous controls for monitoring efficiency of miRNA isolation and reverse transcription steps. Furthermore, we present a novel method for the identification of optimal normalisers with the advantages of assessments of a greater number of potential normalisers (7 in this study, up to 11 with acceptable computational efficiency), clarity in evaluation basis and the ability for end users to weight that evaluation according to their judgement of the relative importance of the features comprising the score. Finally, we indicate 7 normalisers, the combination of which is stable in patients with AD as well as in healthy control subjects, in males and females, of an ageing population.

## Results

We analyzed the levels of selected miRNAs in the plasma samples from 140 subjects, including 27 healthy blood donors and 113 subjects from two independent clinical cohorts consisting of nondemented subjects, subjects with SCI, and patients at early and later AD stages (prodromal AD i.e. MCI-AD, and AD), patients with MCI-non AD, and patients with other forms of dementia (OD) (Table 1).

**Table 1:** Characteristics of study subjects.

Cohort	Disease group	Female			Male		
		n	Age (SD)	MMSE (IQR)	n	Age (SD)	MMSE (IQR)
Bialystok	Cognitively normal (HC)	12	68.8 (7.7)	27 (26.8–28.3)	8	67.0 (10.5)	29.5 (28.8–30)
	Alzheimer’s disease (AD)	32	71.9 (8.4)	21 (18.8–23.3)	8	72.3 (13.4)	23 (21.3–25.3)
Warsaw	Blood donors (non-hospital group)	27	38.8 (11)	–	23	39.8 (10.4)	–
	Subjective cognitive impairment (SCI)	3	64.7 (4.7)	28.7 (28–30)	3	53 (2.7)	28 (27–29)
	Mild cognitive impairment due to AD (MCI-AD)	2	71 (5.7)	25.5 (24.8–26.3)	2	59 (9.9)	28 (27.5–28.5)
	Mild cognitive impairment-non AD (MCI-non AD)	5	56.4 (8.8)	28.3 (28–29.3)	7	55.3 (16.1)	26.7 (24.5–28)
	Alzheimer’s disease (AD)	2	57.5 (10.6)	17.5 (15.8–19.3)	2	61.5 (7.8)	14.55 (11.8–17.3)
	Other dementia (OD)	3	56.3 (7.6)	25 (23.5–26.5)	1	61	27

In these plasma samples, we tested the stability and applicability of 4 exogenously added (spike-in) miRNAs as controls for RT-qPCR and 7 endogenous miRNAs as normalizers (Table 2). Selection of miRNAs was based on the review of the commonly used spike-in controls in the literature and the recommendations of the RT-qPCR reagents manufacturer. In addition, we verified two approaches for detection of haemolysis and exclusion of haemolysed samples from the analysis. We compared absorbance-based detection of haemoglobin with the widely used haemolysis measure based on the RT-qPCR evaluation of two miRNAs levels: plasma marker miR-23a-3p and red blood cell marker miR-451a. Plasma samples with ΔCq (Cq of miR-23a-3p–Cq of miR-451a) < 7 for these two miRNAs are considered clear of contamination while with ΔCq > 7 are considered contaminated^21^.

**Table 2:** A panel of miRNAs tested as potential controls for RT-qPCR-based assessment of miRNA levels in human blood plasma.

miRNA	Sequence	
hsa-miR-23a-3p	AUCACAUUGCCAGGGAUUUCC	Haemolysis controls
hsa-miR-451a	AAACCGUUACCAUUACUGAGUU	
hsa-miR-16-5p	UAGCAGCACGUAAAUAUUGGCG	
Spike-In cel-miR-39-3p	UCACCGGGUGUAAAUCAGCUUG	Exogenous controls (isolation control)
Spike-In cel-miR-54-3p	UACCCGUAAUCUUCAUAAUCCGAG	
Spike-In cel-miR-2-3p	UAUCACAGCCAGCUUUGAUGUGC	Exogenous controls (cDNA synthesis control)
Spike-In cel-miR-238-3p	UUUGUACUCCGAUGCCAUUCAGA	
hsa-miR-93-5p	CAAAGUGCUGUUCGUGCAGGUAG	Endogenous controls (normalisers)
hsa-miR-192-5p	CUGACCUAUGAAUUGACAGCC	
hsa-miR-24-3p	UGGCUCAGUUCAGCAGGAACAG	
hsa-miR-126-3p	UCGUACCGUGAGUAAUAAUGCG	
hsa-miR-16-5p	UAGCAGCACGUAAAUAUUGGCG	
hsa-miR-484	UCAGGCUCAGUCCCCUCCCGAU	
hsa-miR-23a-3p	AUCACAUUGCCAGGGAUUUCC	

**Figure 1:**
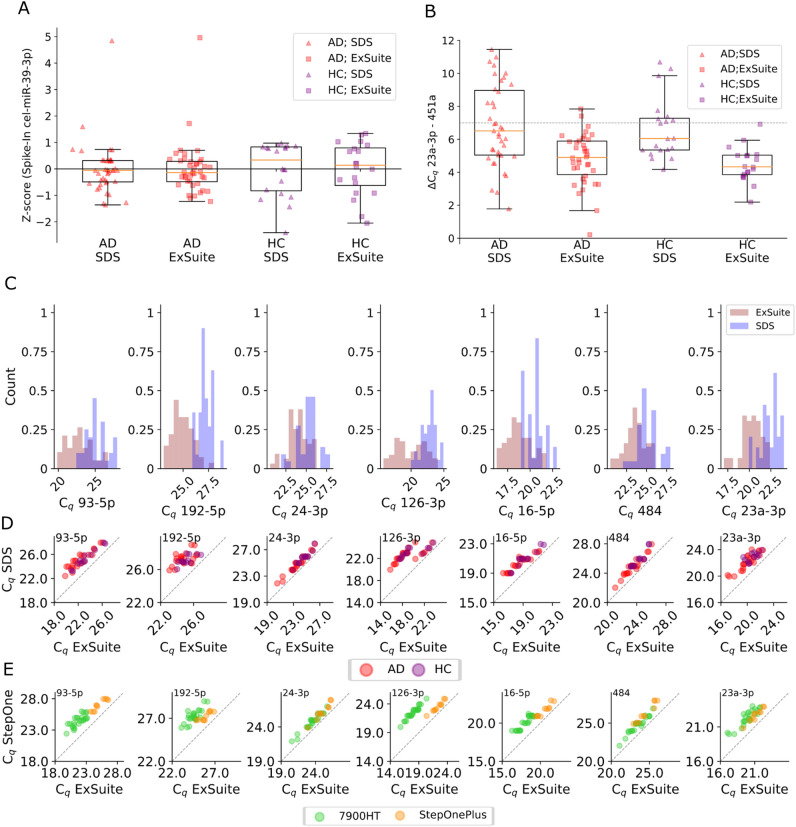
Białystok cohort data analysed using different machines and software. (**A**) Z-score of cel-miR-39-3p spike-in in the group diagnosed with AD and in control group obtained using SDS or ExSuite software. (**B**) Standard evaluation of haemolysis level in the samples based on ΔC_q_ between miR-23a-3p (plasma marker) and miR-451a (red blood cell marker) with the dashed line showing a commonly applied threshold of 7, used as a cutoff for haemolysis. (**C**) Comparison of distribution of C_q_ values of potential normalisers for data analysed with ExpressionSuite (ExSuite) or Sequence Detection System (SDS) Software. (**D**) Pairwise C_q_ plots for each potential normaliser, coloured by diagnostic group: red = Alzheimer’s disease (AD), purple = Cognitively normal (HC). (**E**) Data from D, coloured according to the PCR machine used for analysis: green = 7900HT, orange = StepOnePlus.

Using samples from the Bialystok cohort, we first compared RT-qPCR results obtained for the same sample run in parallel on two different machines, StepOnePlus and 7900HT, and analyzed the results with two different software packages, Sequence Detection System v. 2.4. (SDS, Thermo Fisher Scientific, Waltham, MA, USA) and ExpressionSuite Software v1.3 (Thermo Fisher Scientific, Waltham, MA, USA), respectively. This is a particularly useful validation where the assay is deployed in clinical trials or diagnostic practice. During data processing, it appeared that the SDS software used for analysis introduced some bias into the possible C_q_ values (Supplementary Fig. 1). Moreover, Fig. 1A,B show how differing analysis software influences the number of samples that may be considered for exclusion based on spike-in variability (Fig. 1A) or the widely used haemolysis measure of ΔC_q_ (miR-23a-3p – miR-451a) (Fig. 1B and Supplementary Table 1). This highlights just how crucial it is that any pre-study validation work is fully scrutinised to ensure it is robust before inferences are made relating to areas of study conduct that may render some data unusable. The differences between the analysis software are further exemplified by Fig. 1C and Supplementary Table 2, where the potential normalisers included in the panel show broadly similar distributions between the two software programs but with a consistently lower C_q_ value derived from ExpressionSuite, potentially impacting choices on how samples relate to the upper or lower limits of quantitation. This relationship is supported by Fig. 1D showing no detectable differences between the AD and HC groups, and the scatter plot data consistently lie above the dashed line indicating y = x. Another interesting feature of this view of the data was the clear observation of two discrete populations for miR-126-3p. When replotted using colour to indicate the different machines (Fig. 1E), it becomes obvious that the machines are delivering varied data, which was not detected during the pre-study validation. This is recapitulated for miR-192-5p and miR-16-5p, as well as the other potential normalisers, albeit in a less obvious way. These data clearly demonstrate the variability in RT-qPCR results which can be introduced by machine and software. We thus recommend performing all steps of RT-qPCR analysis, including normalisation, using the same machine and software for the whole study.

**Figure 2:**
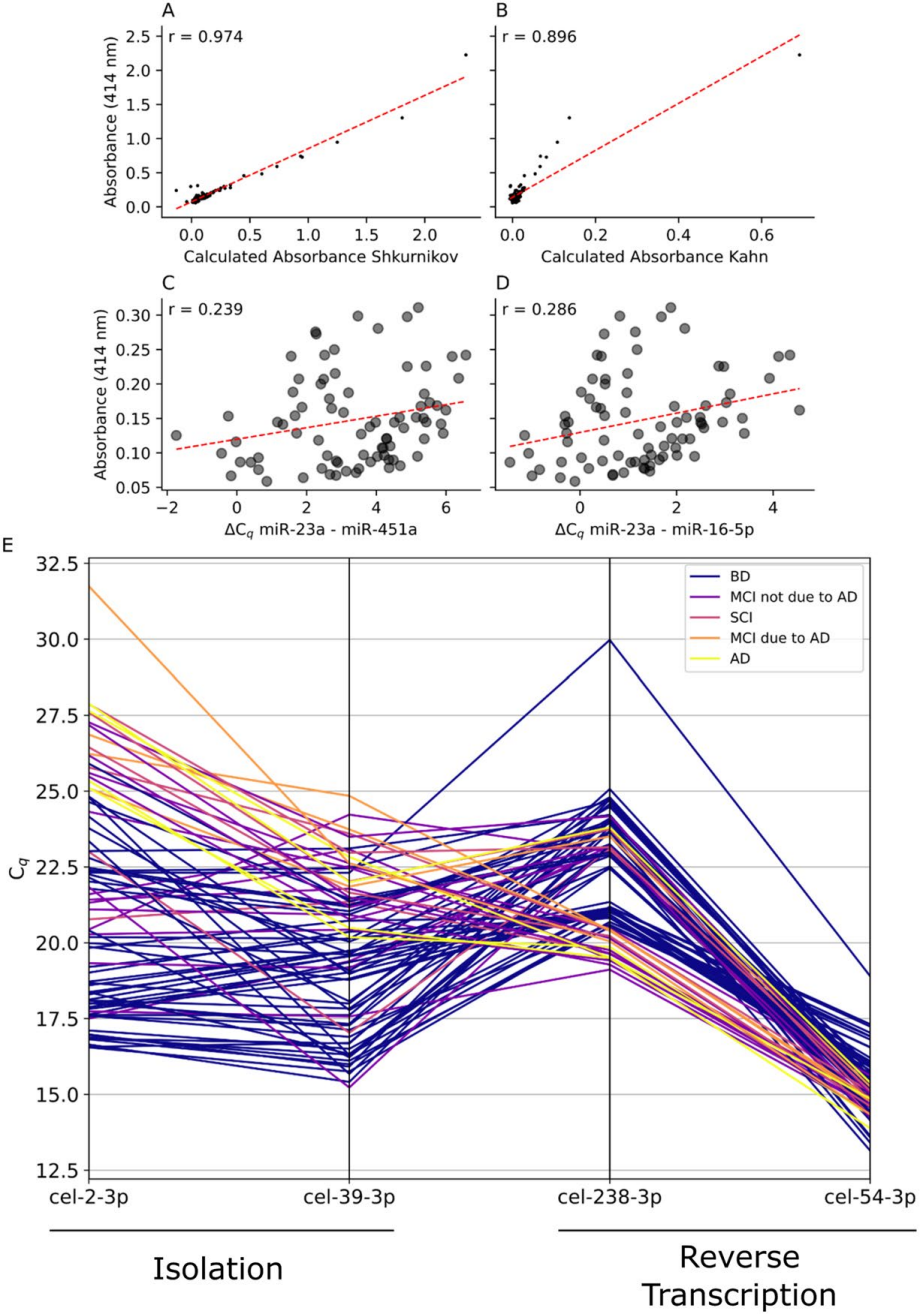
Variability of hemolysis and exogenous controls for miRNA analysis. (**A**–**D**) Four discrete methods are compared to light absorbance at 414 nm, algorithms from Shkurnikov (415, 450 and 700 nm wavelengths)^22^, Kahn (wavelengths 578, 562 and 598 nm)^23^ as well as ΔC_q_ (miR-23a, miR-451a) and ΔC_q_ (miR-23a, miR-16-5p). Linear regression curves (red, dashed) and correlation r values are added to each subplot. (**E**) Parallel axis plot showing C_q_ variability of four added exogenous controls for the Warsaw cohort. Annotation shows controls added prior to miRNA isolation (cel-2-3p and cel-39-3p) and prior to reverse transcription (cel-238-3p and cel-54-3p). Lines coloured according to disease class (*BD* blood donor, *MCI* mild cognitive impairment, *SCI* subjective cognitive impairment, *AD* Alzheimer’s disease).

In the next step using plasma samples from the Warsaw cohort, we further explored the utility of the widely used ΔC_q_ method for assessing haemolysis and compared it with more traditional absorbance-based approaches. Each of the subplots in Fig. 2 shows a different measure of hemolysis plotted against the peak absorbance wavelength for oxyhemoglobin, 414 nm. Compared with even this simple measure, the two plots indicating the ΔC_q_ calculations (Fig. 2C,D) show very poor correlations compared with absorbance-based methods. The upper plots (Fig. 2A,B) use previously published equations^22,23^ combining three different wavelength measurements for which correlations to absorbance (414 nm) are very strong. In Supplementary Fig. 2, we show a t-distributed stochastic neighbor embedding (tSNE), subsequent to a 30 parameter PCA, which is derived from the entire absorbance spectrum for the samples, suggesting that this could be a viable alternative method that would not rely on a priori selection of important wavelengths.

Figure 2E shows the high level of sensitivity of isolation controls to stochastic and sample-specific features compared with those added prior to reverse transcription. On closer inspection, the measurement of cel-238-3p also exhibits a partitioning of measurements into two subgroups (the basis of which could not be determined) but is not related to age, sex or disease status (Supplementary Figs. 3 and 4).

The analysis of normalizer stability in the ageing population comprising healthy controls and AD patients was performed in plasma from the Białystok cohort. Based on these data, we developed a novel method, called BestmiRNorm, to identify the optimal combination of normalisers (http://www.github.com/AndrewWant/BestmiRNorm). The basic premise for our novel method rests on two assumptions: the best combination of normalisers is that which produces the most stable value for the potential normalisers, and the optimal combination may not necessarily be the combination of the single normalisers identified through other methods.

**Figure 3:**
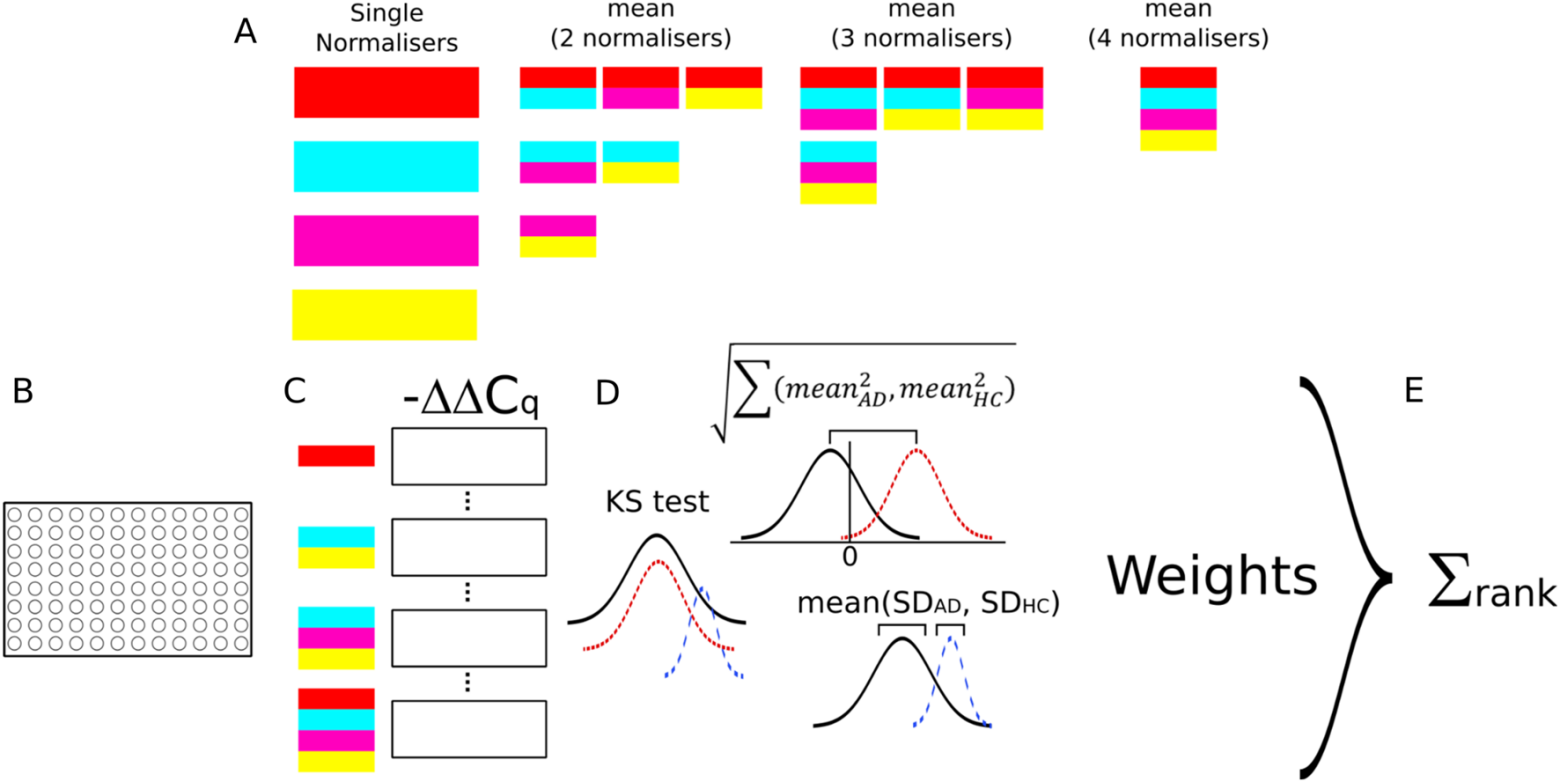
Scheme for the identification of optimal normalisers. (**A**) Representation of the combinatorial approach to evaluating normaliser combinations. For n normalisers, there are 2^n^ − 1 combinations. (**B**) Plasma samples analyzed by RT-qPCR and ExpressionSuite. (**C**) C_q_ values used to calculate − ΔΔC_q_ for each combination of normalisers (abbreviated here). (**D**) Normalised values of each combination of n normalisers for Alzheimer’s disease (AD) and control (HC) groups compared along three metrics—Kolmogorov–Smirnov (KS) test, mean of absolute deviation of group means from zero, and mean of group standard deviations. Data are then ranked by minimising each metric, with the top-ranked normaliser combination given n_combinations_ points (15 in the example above), and the bottom given 0. Where desired, weights can be applied to each scoring component by multiplying the rank score by a specified value. (**E**) Individual rank scores for the metrics are summed, and then all normaliser combinations are ranked according to that sum.

Our approach was to calculate − ΔΔC_q_ for each normaliser/combination (Fig. 3A–C) and then score the data on the specified metrics (Fig. 3D). Once the rankings have been assigned, weights can be applied to each of the metrics to amplify its rank value, according to the judgement of importance by the experimenter (Fig. 3E). Following this, the individual weighted metrics can be summed, with a final ranking used to evaluate the best normaliser combination.

**Figure 4:**
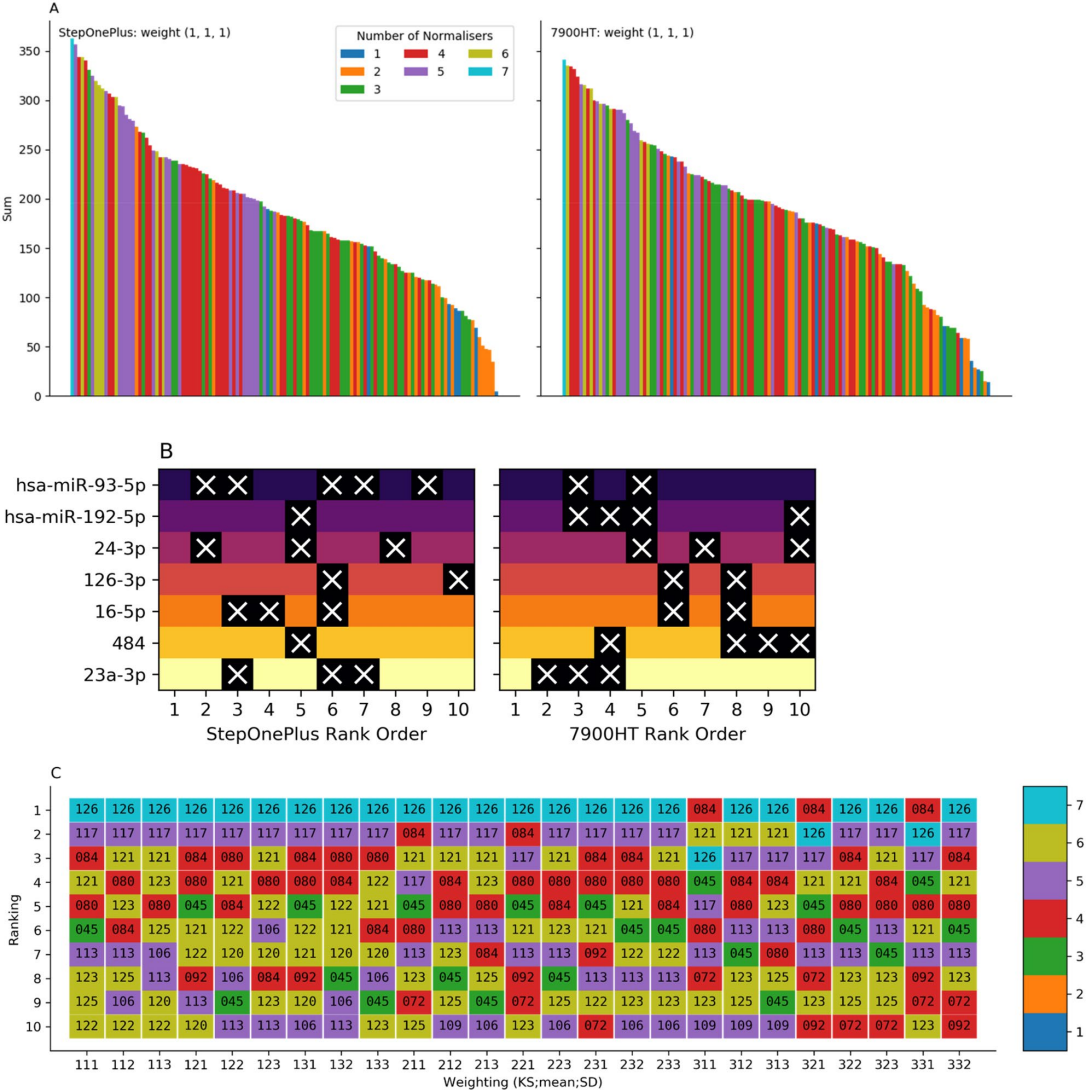
Normaliser identification in samples from the Białystok cohort. (**A**) Each vertical stripe represents a specific combination of normalisers with equal weighting applied to the score components (left: StepOnePlus, right: 7900HT). Colour indicates the number of normalisers in the given combination. (**B**) Top 10 ranked normaliser combinations with equal weighting for StepOnePlus (left) and 7900HT (right). Each row shows a specific normaliser, with a white cross showing which ranks that normaliser is absent from. (**C**) Influence of weighting on the total score ranking of normaliser combinations for StepOnePlus. Up to threefold weighting (222 and 333 are omitted) was applied to the scoring components. Numerical annotation indicates the substituted code for the combination (see Supplementary Table 3 for translation), and colour shows the number of normalisers used.

With equal weighting applied to the scoring metrics, Fig. 4A shows the comparison of ranked scores for the two different qPCR machines used (see Supplementary Fig. 5A–Y for all calculated weightings). The samples analysed on the StepOnePlus appear to show considerably more clustering of ranked scores, with noticeably large single-colour blocks, compared with 7900HT. Figure 4B illustrates the specific normalisers found in the top 10 ranked combinations, where miR-192-5p and miR-484 are present in 9 of the 10 top-ranked combinations, suggesting that these are particularly stable. The application of weights to the scores (Fig. 4C) suggests that the mean of all 7 potential normalisers is broadly stable, with the largest deviation from this pattern observed when a large weight is applied to the KS score. The full conversion table for the code numbers is included in Supplementary Table 3, from which it can be seen that the other notable miR combinations with consistent performance are 117 (hsa-miR-192-5p, 126-3p, 16-5p, 484, 23a-3p) and 84 (hsa-miR-192-5p, 24-3p, 126-3p, 484).

**Figure 5:**
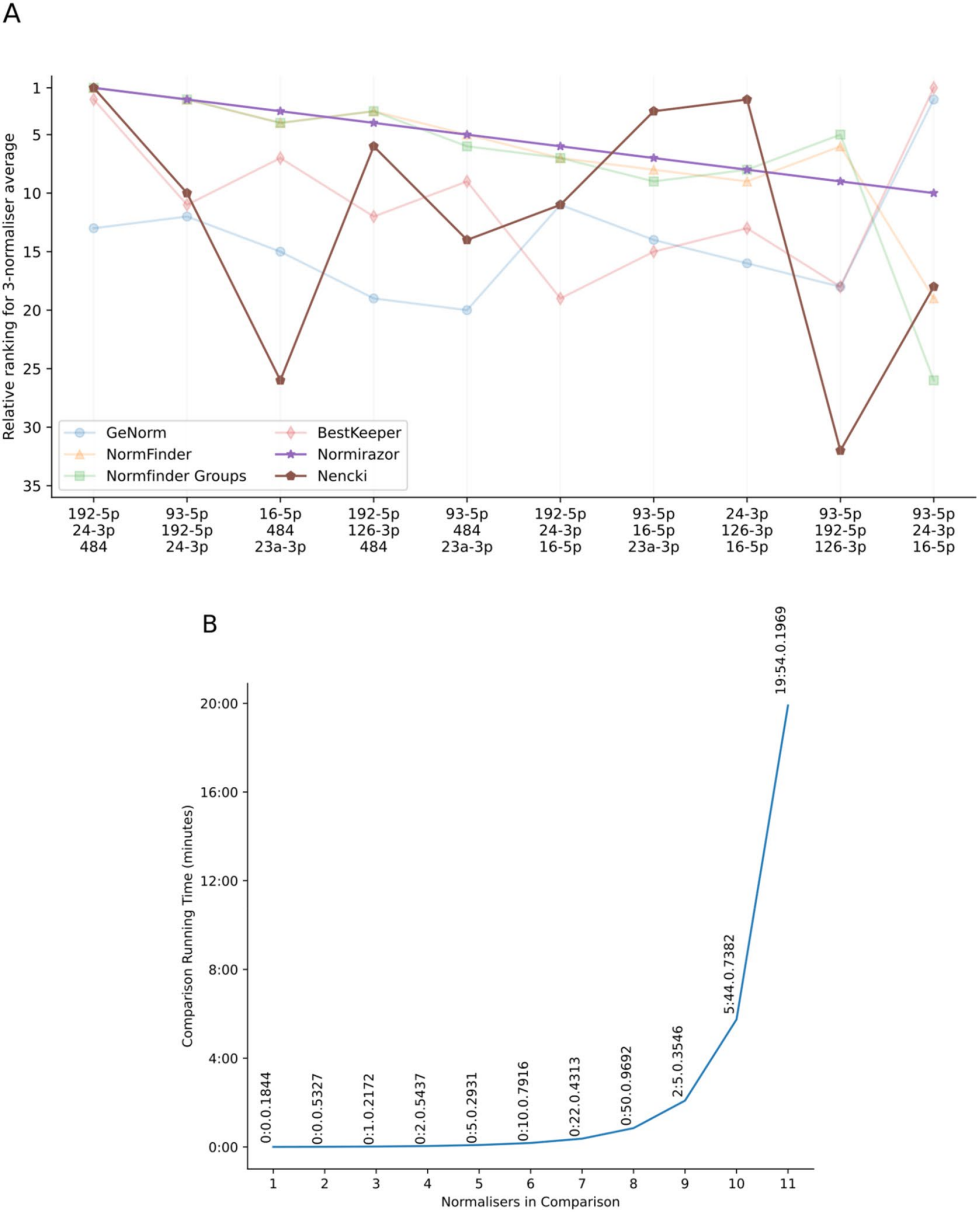
Białystok cohort normaliser identification comparison. (**A**) Comparison of the relative ranking of the top ten 3-normaliser combinations using Normirazor (purple star), with its constituents (blue circle, orange triangle, green square and red diamond) and BestmiRNorm (Nencki, brown pentagon). (**B**) Computational running time (minutes) for evaluating the ascending total number of normalisers in combination.

Figure 5A shows the comparison of the top ten ranked combinations of three normalizers according to the most widely used algorithms and our novel approach. The most recent publication, Normirazor (purple star), was used as a reference for the top ten most stable combinations. It can clearly be seen that our BestmiRNorm method is distinct from Normirazor, which in turn appears to be dominated by Normfinder. Interestingly, both our method and Normirazor found the same optimal combination of 3 normalizers (hsa-miR-192-5p, 24-3p, 484), despite the general disagreement between methods.

Figure 5B shows the computational running time for our algorithm when comparing increasing numbers of normalizers. The current computational implementation adds an approximately 2.5-fold increase in time (minutes), and 11 normalizers can be compared in only 20 min of computational time, a significant improvement over current standard approaches.

## Discussion

Blood is one of the most readily accessible matrices for diagnostic testing, and plasma circulating miRNAs show very promising features as biomarkers for many aging-related diseases, including AD. Despite considerable research effort, none of the plasma circulating miRNAs identified as candidate biomarkers have yet been broadly verified and implemented in clinical practice. One of the main reasons for this lack of implementation is the variation in RT-qPCR controls and normalization approaches for miRNA profiling across different studies.

RT-qPCR is widely used due to its specificity and sensitivity, small amount of template RNA required, easily available kits and instrument platforms, and relatively low costs of assays. While RT-qPCR serves as an invaluable tool for examining miRNA levels in plasma, it is crucial to implement proper normalization to prevent data misinterpretation. A sound normalization strategy stands as one of the fundamental components outlined in the MIQE Guidelines (Minimum Information for Publication of Quantitative Real-Time PCR Experiments)^24^. However, so far no optimal normalization strategy for RT-qPCR based miRNA profiling exists and verified guidelines specifically addressing the quantification of circulating miRNAs based on RT-qPCR are not available. The necessity for standardized normalization approaches and standardization of all steps in RT-qPCR-based miRNA profiling in plasma has been widely acknowledged in search for potential biomarkers in various pathologies including neurodegeneration^5,25,26^.

The identification of miRNAs meeting normalizer criteria has proven challenging. It is now recognized as optimal to normalize qPCR data using biomolecules of the same class^18^. However, profiling studies have uncovered significant variability in miRNA expression within various tissues and body fluids, with their blood levels changing during aging or in pathologies. Consequently, no single miRNA can serve as a “universal” normalizer^27,28^. It emphasizes the importance of verifying the stability of selected normalizers in the specific tissue or body fluid of interest in each study.

To facilitate this task, our study presents a set of validated normalizers in human plasma for aging and Alzheimer’s disease, along with the newly validated algorithm BestmiRNorm as normalization software. The miRNAs confirmed as stable normalizers in this study are recommended as prime candidate normalizers for other studies on miRNA profiling via RT-qPCR, in conjunction with the BestMiRNorm computational method to validate their stability in a particular cohort of interest. These tools can assist researchers in confirming miRNAs as normalizers for RT-qPCR-based miRNA profiling in their studies.

Furthermore, we examined common analytical pipelines for profiling circulating miRNAs and we propose an optimization of current standard practice at key steps that may introduce variability. The absence of such a unified comprehensive workflow for standardizing RT-qPCR-based miRNA profiling in human plasma has impeded progress in developing miRNAs as plasma biomarkers thus far.

**Figure 6:**
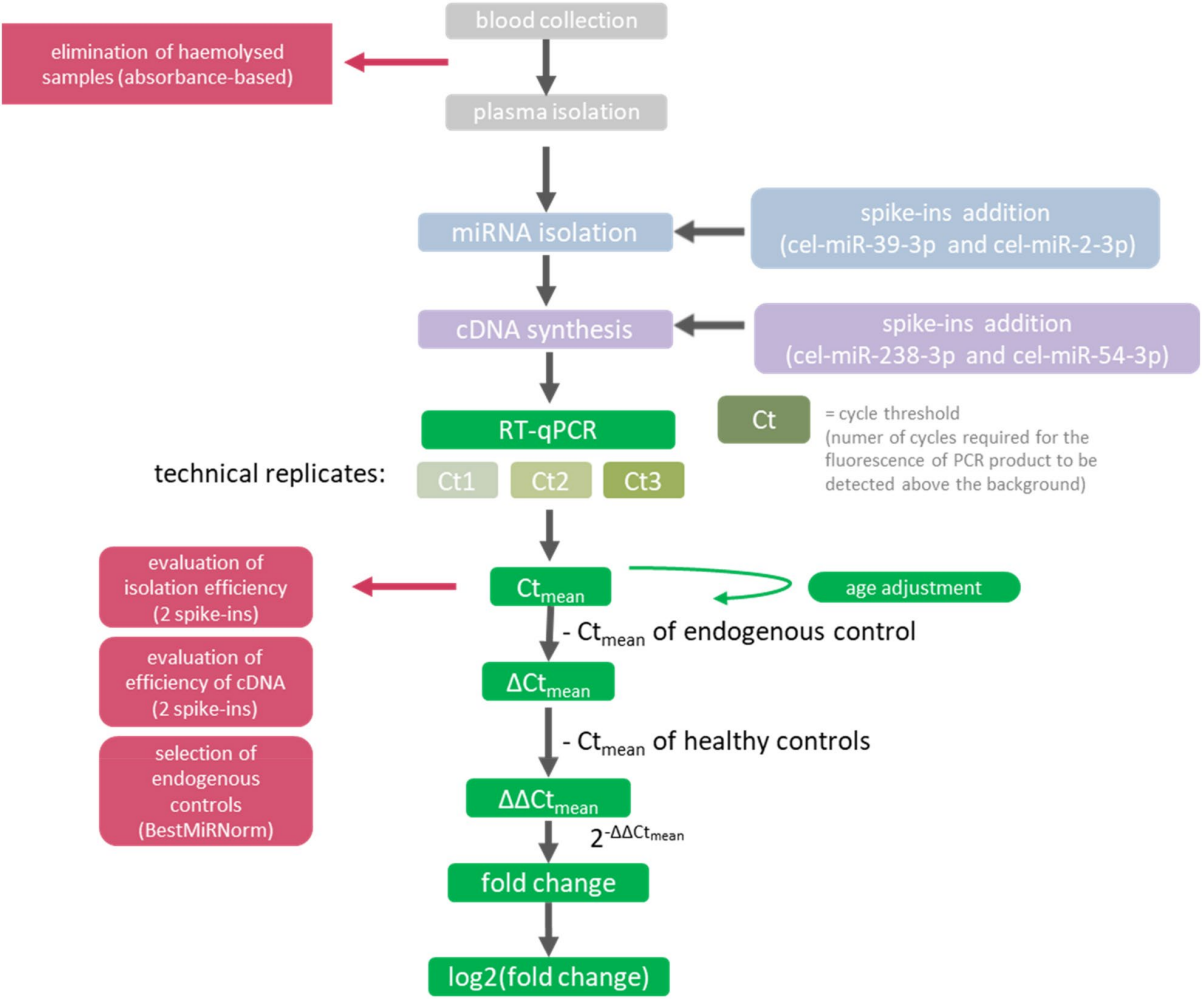
Flowchart of the optimal study design. A flow chart describing the recommended subsequent steps of plasma-derived miRNA analysis by RT-qPCR.

Our recommendations concern the following qPCR steps: homogeneous condition of sample collection and storage and elimination of haemolysed samples prior to analysis, addition of exogenous miRNAs as controls of miRNA isolation and reverse transcription, and the normalisation approach. We have summarized our recommended standardized methodology for obtaining reliable and comparable miRNA analysis data in blood plasma (and other body fluids) by RT-qPCR in Fig. 6.

After storage, the first necessary step in the analysis of miRNA from blood serum or plasma is to assess sample quality by evaluating haemolysis (Fig. 6). It has long been known that haemolysis can profoundly influence the measured values of a broad range of miRNAs^29–31^. The selection of appropriate methodologies to test for haemolysis is a critical step to assess blood sample quality and identify true changes in miRNA expression levels in this body fluid^21^. The most commonly used method for haemolysis evaluation is based on the ratio of specific miRNAs, usually miR-23a to miR-451a or to miR-16-5p, with samples exceeding a prespecified threshold (usually 7) excluded from the analysis. However, the main outcome of our study challenges the accepted standard practice of examining only this miRNA ratio. Our experiments clearly demonstrated that this approach did not work properly when compared with haemoglobin absorbance measurement. The other groups have also indicated problems with false positive results of haemolysis assessment by specific miRNAs ratio in comparison to alternative methods, e.g. absorbance-based ones^32^. It has been shown that the traditional absorbance-based approach for assessing haemolysis has limitations due to haemoglobin absorbance interferences from analytes (e.g. bilirubin, lipids) and other molecules presented in a sample. However, recent studies on haemolysis focus on developing and improving absorbance methods by measurement of few different wavelengths and additional mathematical algorithm for interference determination and elimination. Increasing number of reports on lysis quantification in blood sample have confirmed the effectiveness of using absorbance-based method improved by mathematical algorithm^22,23,33–35^. Interestingly, we have also shown that usage of the entire absorbance spectrum for the samples to evaluate haemolysis could be a viable alternative method that would not rely on a priori selection of important wavelengths (Supplementary Fig. 2). There are also alternative haemolysis assays proposed for blood sample experiments, such as monitoring of haemolysis in real-time based on resistance measurement^36^ or optofluidic waveguide sensor^37^ and haemolysis detection in silico^38^. Nevertheless, these methods are not well-studied and verified and have not been compared in our study. Thus, to avoid errors in miRNA quantification, we suggest an absorbance-based method as the preferred option for assessing haemolysis. Additionally, a visual pre-check of the haemolysis status can also be useful.

Secondly, our studies indicate the necessity of using at least two exogenous controls to evaluate the efficiency of each of the two steps: miRNA isolation and reverse transcription (Fig. 6). Typically, an exogenous control in the form of a synthetic spike-in miRNA is added at a known concentration before isolation or RT to demonstrate the efficiency of each process/reaction, allowing for correction or elimination of unreliable samples. We recommend using spike-in controls for both steps, with more than one spike-in for each step. This double control for each step of miRNA processing is necessary because, despite the synthetic origin of spike-ins, the isolation and reverse transcription of each individual miRNA spike-in may be differentially affected by other components of the plasma sample. Using spike-in miRNAs as controls of these two steps has advantages compared to endogenous reference miRNAs. Endogenous miRNAs are affected similarly to the target miRNAs by experimental conditions because of the matched starting physiological context. However, variations in technical conditions of isolation and RT of specific miRNAs can lead to a discontinuity between certain endogenous controls and target miRNAs in particular samples. As such, the benefit of simultaneous use of endogenous normalisers with exogenous controls (spike-ins) to enhance the quality and reproducibility of experimental findings is essential.

Thirdly, we propose here a novel and transparent method called BestmiRNorm for selecting optimal normalisers in an ageing population for proper RT-qPCR data analysis openly accessible at github.com/AndrewWant/BestmiRNorm. Moreover, we verified stability of plasma levels of 7 candidate normalisers in a non-demented ageing population and in the subjects at early and later AD stages and recommend their application in search for circulating miRNAs biomarkers in ageing-related diseases such as AD. Variability in normalization approaches is the most important factor responsible for the low reproducibility of plasma circulating miRNAs identified by qPCR as biomarkers^5^. Our review of the literature shows that no universal endogenous normalisers are used, and the applicability of reference genes from other studies or the use of a single reference gene does not guarantee reliable miRNA quantitation^39^.

BestmiRNorm, our novel approach for identifying optimal normalisers, enables comparison of up to 11 normalisers in combination, in a computationally efficient fashion. Furthermore, uniquely to our method, the user can define what a “good” combination of normalisers means to them through the application of weighting to the scores for overall variance, average net mean deviation from zero and/or distribution of two classes of interest.

Finally, the logic behind the evaluation of normalizers is clearer and more transparent, providing users and the audience of resulting publications with evident justification for the "best" normalizers. These results emphasize the necessity of increasing standardization and analytical transparency to advance miRNAs as therapies or markers of disease. Furthermore, we provide an open-source tool that enables other researchers to apply our methods to their own data and extend and improve our approach.

In conclusion, to avoid misleading conclusions regarding circulating miRNAs as candidate biomarkers and to facilitate comparison of miRNA biomarkers across studies and laboratories for their translation from the discovery phase to clinical diagnostics, standardization of the preanalytical and analytical methods is necessary. In this study, we propose an optimized analytical methodology for obtaining reliable and comparable miRNA analysis data in blood plasma using RT-qPCR. Our studies indicate the necessity of:

- evaluating the quality of the sample and excluding haemolysed samples based on absorbance measurements,
- using at least two exogenous controls for miRNA isolation and reverse transcription, and adjusting/correcting for the efficiency of these steps, and
- selecting optimal normalizers for data analysis using our novel, openly accessible, and computationally efficient method called BestMirNorm.

Using the BestMirNorm method, we successfully selected and validated seven stable normalizers in an aging population. We recommend their use in biomarker assays of miRNAs in aging-related diseases.

## Methods

### Reagents

miRNeasy Serum/Plasma Advanced Kit (Qiagen, Hilden, Germany, # 217204); TaqMan^®^ Advanced miRNA cDNA Synthesis Kit (Applied Biosystems, Bedford, MA, USA, #A28007); TaqMan^®^ Fast Advanced Master Mix (Applied Biosystems, Bedford, MA, USA, # 4444556); TaqMan^®^ Advanced miRNA Assays Single-tube assays (Applied Biosystems, Bedford, MA, USA, # A25576: 478293_mir, Spike-In cel-miR-39-3p; 478291_mir, Spike-In cel-miR-2-3p; 478292_mir, Spike-In cel-miR-238-3p; 478410_mir, Spike-In cel-miR-54-3p; 478262_mir, hsa-miR-192-5p; 478210_mir, hsa-miR-93-5p; 477860_mir, hsa-miR-16-5p; 478308_mir, hsa-miR-484; 478532_mir, hsa-miR-23a-3p; 478107_mir, hsa-miR-451a; 477992_mir, hsa-miR-24-3p; 477887_mir, hsa-miR-126-3p; 478369_mir, hsa-miR-29b-3p; 478007_mir, hsa-miR-30b-5p; 478048_mir, hsa-miR-34a-5p; 477885_mir, hsa-miR-125b-5p; 478581_mir, hsa-miR-135a-5p; 477910_mir, hsa-miR-142-3p; 478399_mir, hsa-miR-146a-5p; 478490_mir, hsa-miR-200a-3p; 478432_mir, hsa-miR-483-5p; 478128_mir, hsa-miR-486-5p, 478348_mir, hsa-miR-502-3p; 477952_mir, hsa-miR-191-5p).

### Human subjects

Plasma samples were collected from Polish subjects of two independent cohorts enrolled at the Medical University of Bialystok (Bialystok cohort) and at Central Clinical Hospital of the Ministry of Interior (MSWiA) in Warsaw (Warsaw cohort). Plasma collection and analyses were performed in accordance with the Declaration of Helsinki after obtaining approval from Bioethical Committees at the respective centres (Medical University of Bialystok: R-I_002/459/2018 and MSWiA in Warsaw: 106-2016). Patients were diagnosed with AD, mild cognitive impairment due to AD (MCI-AD), MCI not due to AD (MCI-non AD), or subjective cognitive impairment (SCI), according to the National Institute of Neurologic, Communicative Disorders and Stroke/Alzheimer’s Disease and Related Disorders Association (NINCDS-ADRDA) criteria and recommendations from the National Institute on Aging-Alzheimer's Association workgroups^1^. Diagnosis was based on clinical interview and the MMSE test (global cognitive impairment assay, scale 0–30) and was additionally supported by the assays of standard AD biomarkers in the cerebrospinal fluid: Aβ peptides, t-tau (“total” tau protein) and p-tau-181 (tau protein phosphorylated at threonine 181). The CSF assays were carried out in the hospital laboratory using an ELISA kit (Innogenetics, Gent, Belgium). Peripheral blood samples were collected after written informed consent was obtained from all study subjects or their legal representatives. Inclusion criteria for samples were clinical diagnosis of AD, MCI-AD, MCI-non AD, SCI or cognitively normal. The exclusion criteria were as follows: an age below 50, identified comorbidities such as depression, other dementia or neurologic disease, a history of alcohol or drug abuse, and other serious medical conditions that might influence cognition. The sample analysis was performed in a blinded manner to limit the possibility of bias.

All protocols for CSF collection and CSF assays were recommended by the international JPND BIOMARKAPD consortium and adhere to the most recent guidelines^40^. A control group of samples from healthy blood donors from the Warsaw blood donation service was also included. The characteristics of the study subjects are presented in Table 1.

### Plasma collection

Blood was collected by venipuncture into BD Vacutainer K2-EDTA tubes. The samples were immediately centrifuged, and aliquots of plasma were stored in nuclease-free tubes at − 80 °C. The procedure was carried out in adherence to the guidelines for the standardization of preanalytic variables for blood-based biomarker studies in Alzheimer's disease research of the international working group^41^ and to the detailed SOPs (Standard Operating Procedures) established under the international European Union Horizon 2020 FET OPEN grant 737390 “ArrestAD” (Warsaw cohort) and 7th European Union Framework grant 2/BIOMARKAPD/JPND/2012JPND (Bialystok cohort).

### miRNA isolation

miRNA was isolated using the miRNeasy Serum/Plasma Advanced Kit (Qiagen, Hilden, Germany, # 217204) according to the manufacturer’s recommendation. The spike-in cel-miR-39-3p (Białystok cohort) or cel-miR-39-3p and cel-miR-2-3p (Warsaw cohort) were added at a fixed amount per isolation (after lysis stage). Target sequences of exogenous controls (spike-ins) are included in Table 2.

### RT-qPCR

Synthesis of cDNA and RT-qPCR were performed according to TaqMan^®^ Advanced miRNA Assays Single-tube assays (Applied Biosystems, Bedford, MA, USA, # A25576). miRNA was reverse transcribed with a TaqMan^®^ Advanced miRNA cDNA Synthesis Kit (Applied Biosystems, Bedford, MA, USA, #A28007) according to the manufacturers’ protocols (with 2 µL of sample eluent) using a Master cycler nexus gradient (Eppendorf, Hamburg, Germany) and T100™ Thermal Cycler (Bio-Rad, Hercules, USA). The cDNA synthesis controls (cel-miR-238-3p and cel-miR-54-3p) were added to evaluate the efficiency of this reaction. Target sequences of exogenous controls (spike-ins) are included in Table 2.

RT-qPCR was performed according to the manufacturer’s protocol using a TaqMan^®^ Fast Advanced Master Mix (Applied Biosystems, Bedford, MA, USA, # 4444556) with a 1:10 dilution of cDNA template and thermal profile of 95 °C for 20 s; 95 °C for 1 s and 60 °C for 20 s in 40 cycles on a StepOnePlus™ Real-Time PCR System (Applied Biosystems, Bedford, MA, USA) or Abi 7900HT (Applied Biosystems, Bedford, MA, USA) as indicated. Data were analyzed using either ExpressionSuite Software v1.3 (Thermo Fisher Scientific, Waltham, MA, USA) or Sequence Detection System (SDS) Software SDS v. 2.4 (Thermo Fisher Scientific, Waltham, MA, USA).

### Absorbance measurements of plasma

A wavelength scan (220–750 nm) was performed on 2 µL isolated plasma using a NanoDrop 2000 (Thermo Scientific™, Waltham, MA, USA).

### Normaliser identification

The Normirazor webtool^20^ was used to evaluate candidate normalizers up to 3n combinations, providing outputs from Normfinder, Bestkeeper and Genorm as well as the Normirazor aggregated score. Our new method called BestmiRNorm for identifying normalisers was written in the Python programming language (v. 3.7.3), using pandas (v. 1.2.3), numpy (v. 1.17.1), scipy (v. 1.3.1). The method we developed uses three measures to evaluate the miRNA C_q_ values for potential normalisers: Kolmogorov–Smirnov score (KS-score), average displacement from a mean of zero and mean standard deviation. In each case, the comparators are two distinct biological groups of interest (such as Alzheimer’s disease and cognitively normal controls). Before comparison, the group of potential normalisers was normalized according to the log_2_(2^−ΔΔCq^) method^42^. To enable anyone to access, download and run BestmiRNorm on their own data, the code is published on github.com.

### Statistical analysis and software

Raw RT-qPCR data were analyzed using either ExpressionSuite v1.3 (Thermo Fisher Scientific, Waltham, MA, USA) or Sequence Detection System (SDS, Thermo Fisher Scientific, Waltham, MA, USA) software v. 2.4. All C_q_ data were divided into sex groupings and age-adjusted prior to additional analysis (Supplementary Fig. 6A–C and Supplementary Fig. 7).

Statistical analysis was performed using GraphPad Prism 9.1.2 or SciPy (v.1.3.1). PCA and tSNE were carried out using scikit-learn (v. 0.21.3).

### Ethics approval and consent to participate

Human blood plasma collection and analyses were performed in accordance with the Declaration of Helsinki after obtaining approval from Bioethical Committees at the Medical University of Bialystok: R-I_002/459/2018 and at the Central Clinical Hospital of the Ministry of Interior and Administration (MSWiA) in Warsaw: 106–2016. Written informed consent was obtained from all of the patients or their legal representatives.

### Supplementary Information

Supplementary Information.

## Data Availability

The novel normalization method BestmiRNorm is publicly available from http://www.github.com/AndrewWant/BestmiRNorm. Example template files are available from: 10.5281/zenodo.7060403. The datasets generated and used during the current study are available from the corresponding author on reasonable request.

## Abbreviations

miRNA: MicroRNA
RT-qPCR: Reverse transcription-quantitative polymerase chain reaction
Cq: Quantification cycle
AD: Alzheimer’s disease
MCI: Mild cognitive impairment
SCI: Subjective cognitive impairment
HC: Healthy control
CSF: Cerebrospinal fluid, p-tau: phosphorylated tau protein
GAPDH: Glyceraldehyde-3-phosphate dehydrogenase
SOPs: Standard operating procedures
