# Supplementary material for: Optimized RT-qPCR and a novel normalization method for validating circulating miRNA biomarkers in ageing-related diseases
Source: Sci Rep. 2023 Nov 27;13:20869. doi: 10.1038/s41598-023-47971-3 (PMC10682428; doi:10.1038/s41598-023-47971-3)
Supplement: Supplementary file 1 — Supplementary Information. [file 41598_2023_47971_MOESM1_ESM.pdf]

Optimized RT-qPCR and a novel normalization method for validating circulating miRNA biomarkers in ageing-related diseases

Andrew Want1, Karolina Staniak1, Wioleta Grabowska-Pyrzewicz1, Aleksandra Fesiuk1, Karolina Staniak1, Anna Barczak2, Tomasz Gabryelewicz2, Agnieszka Kulczyńska-Przybik3, Barbara Mroczko3, Urszula Wojda1*

*Correspondence to: u.wojda@nencki.edu.pl

Supplementary Data

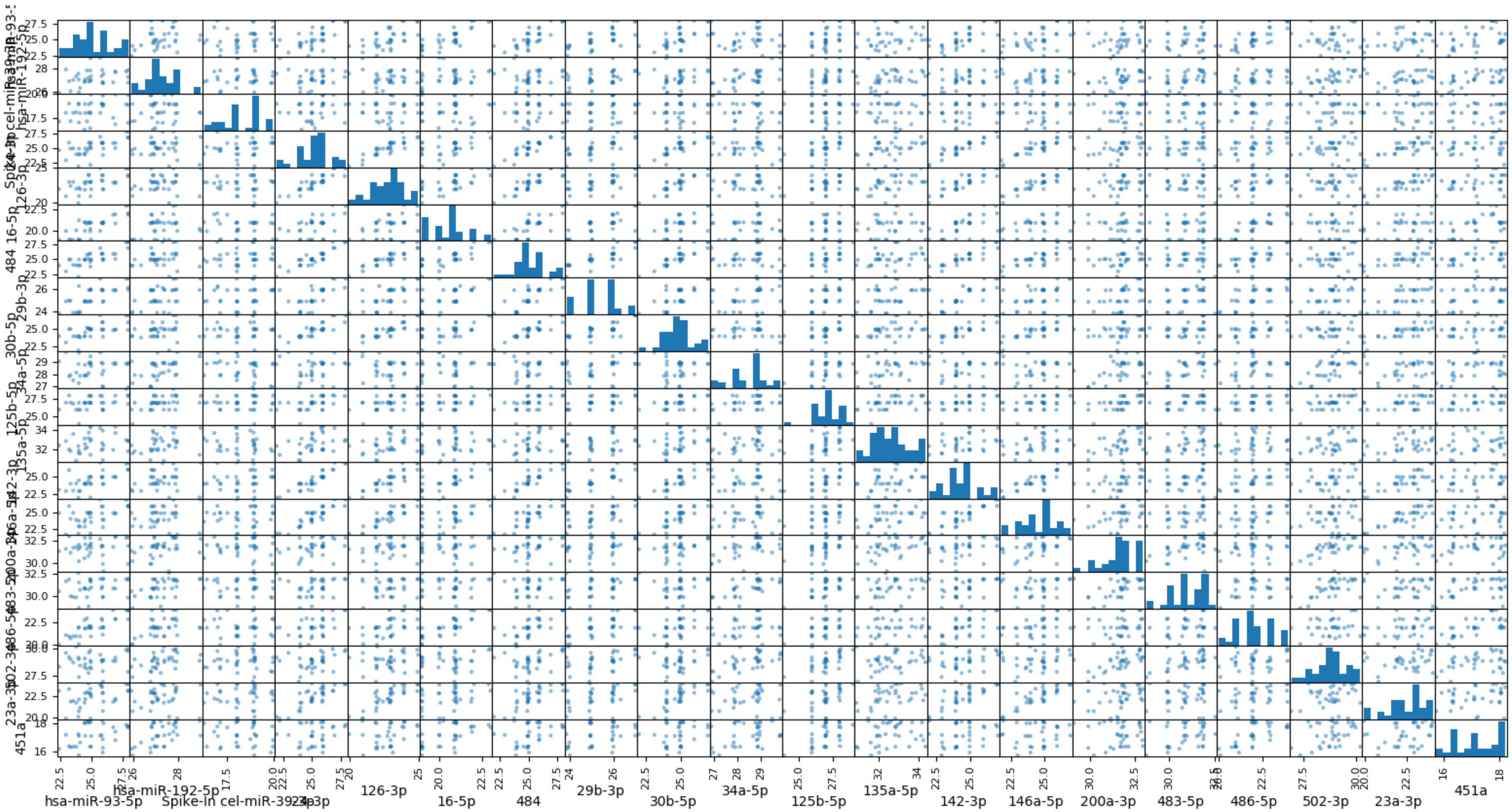

Supplementary Figure 1 Pairwise scatter plots of raw miRNA Cq data from SDS software showing clustering around discrete values.

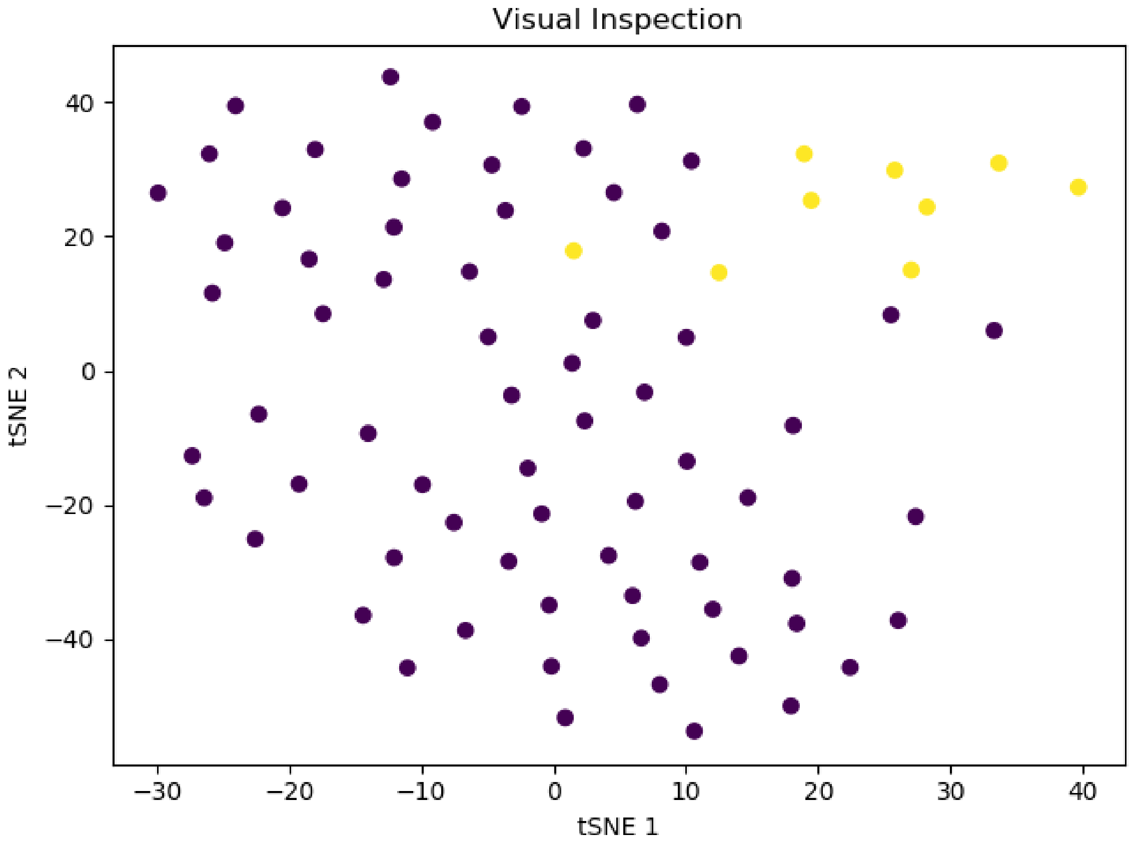

Supplementary Figure 2. t-distributed Stochastic Neighbour Embedding (following PCA for 30 components) of the absorbance wavelengths from 220-750 nm. Colour is according to visual perception of haemolysis in the sample by a human operator (yellow=visibly haemolysed; purple=not visibly haemolysed).

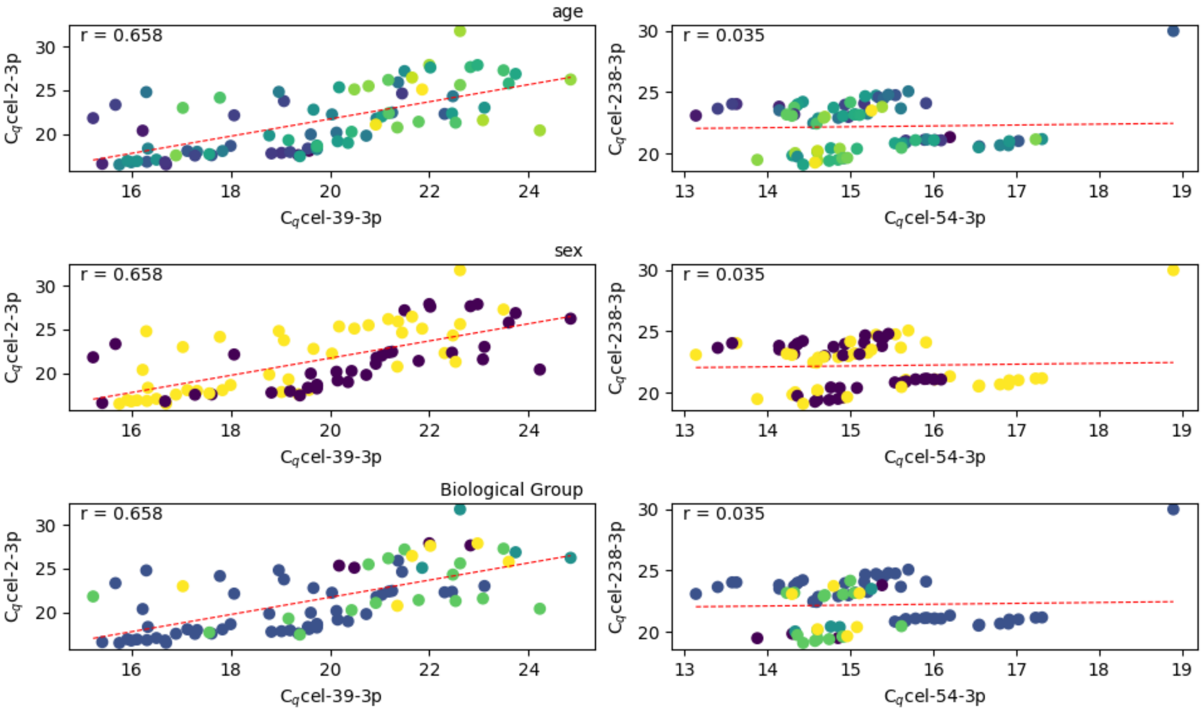

Supplementary Figure 3. Correlation between isolation (left) and reverse transcription (right) exogenous controls. Markers coloured according to age (top), sex (middle) or disease status (bottom).

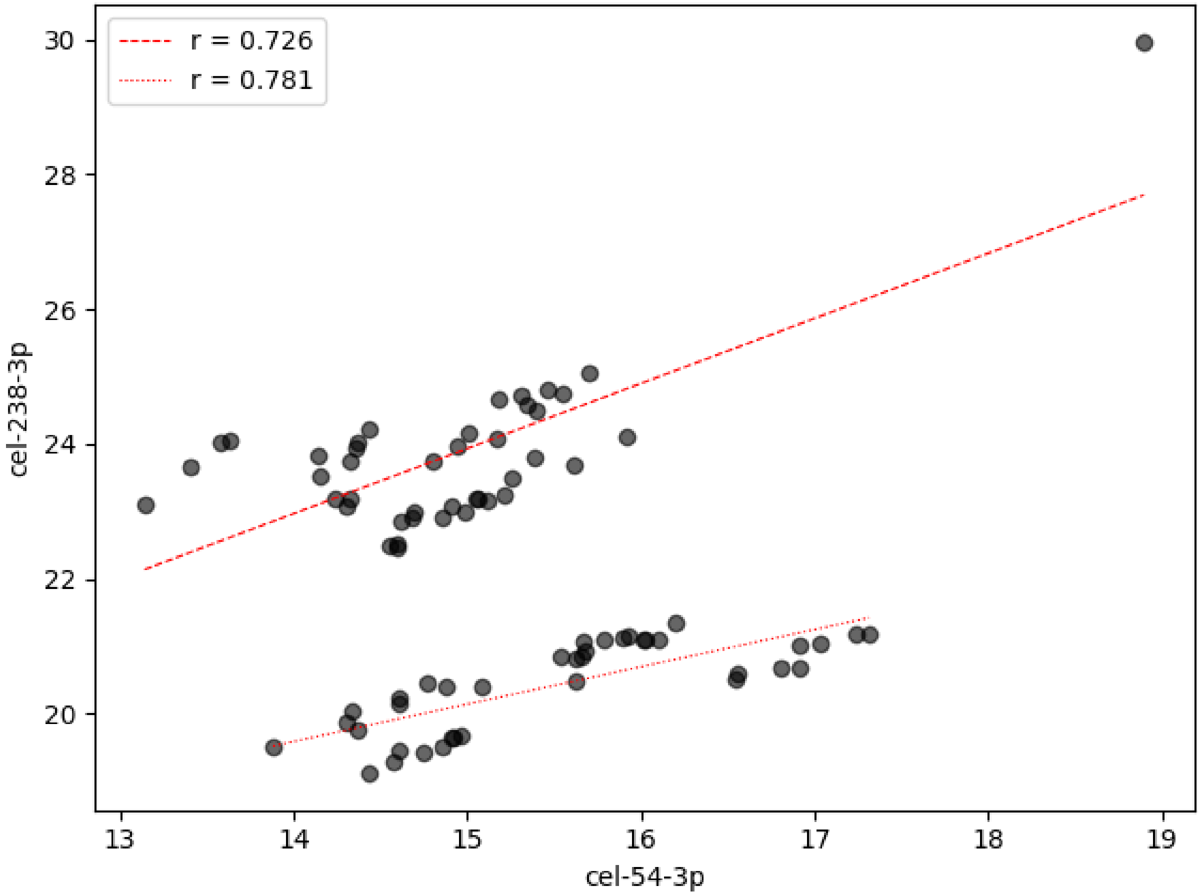

Supplementary Figure 4. Separate correlations of exogenous controls added prior to reverse transcription.

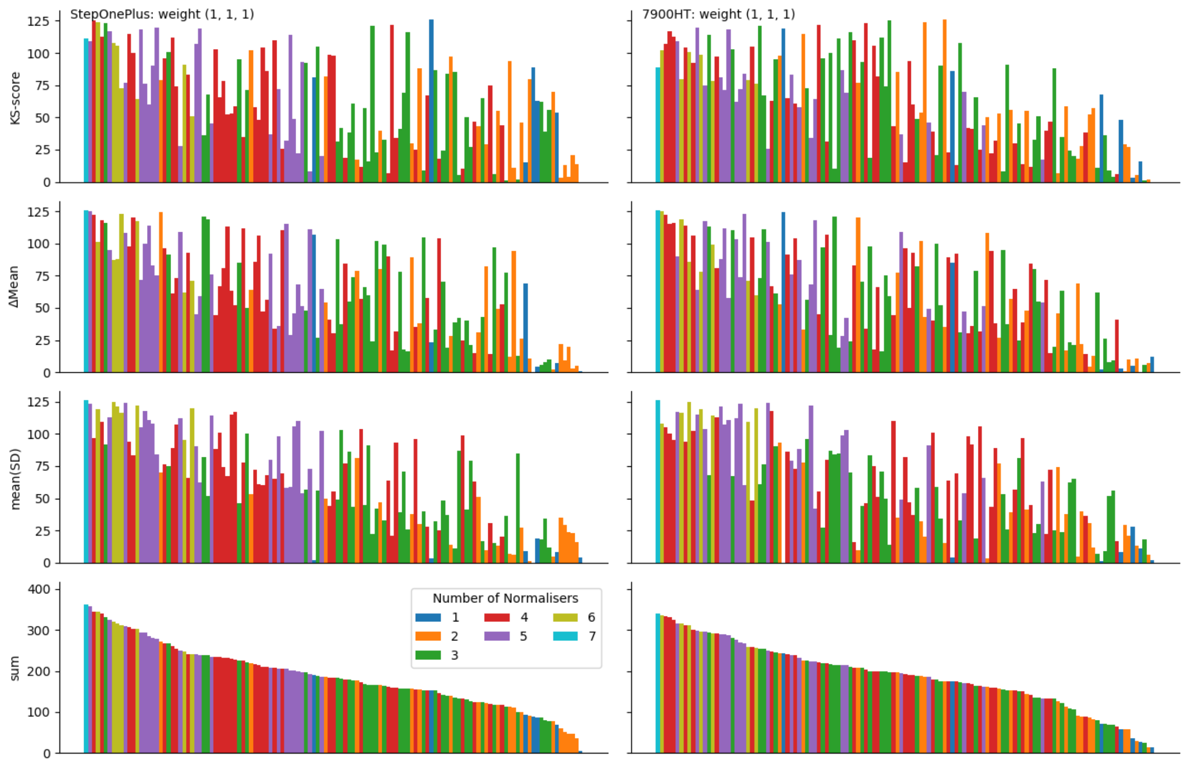

Supplementary Figure 5A. Individual and sum score components with weights 1; 1; 1.

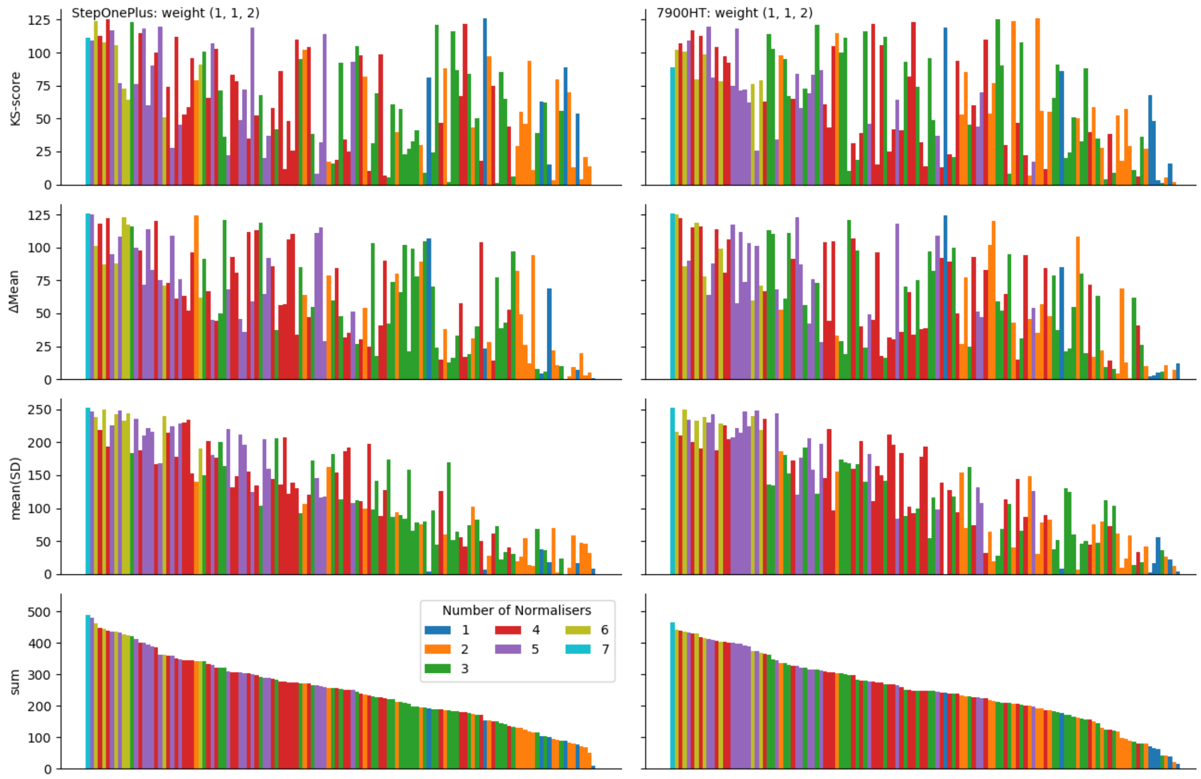

Supplementary Figure 5B. Individual and sum score components with weights 1; 1; 2.

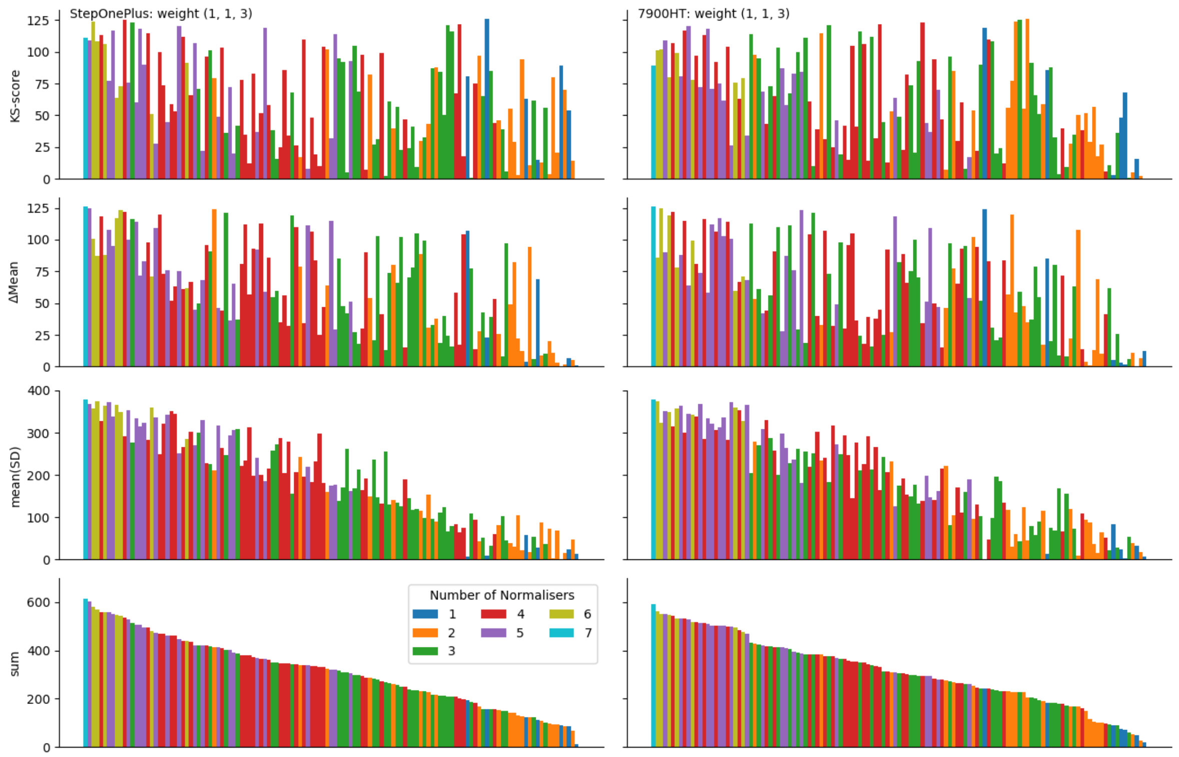

Supplementary Figure 5C. Individual and sum score components with weights 1; 1; 3.

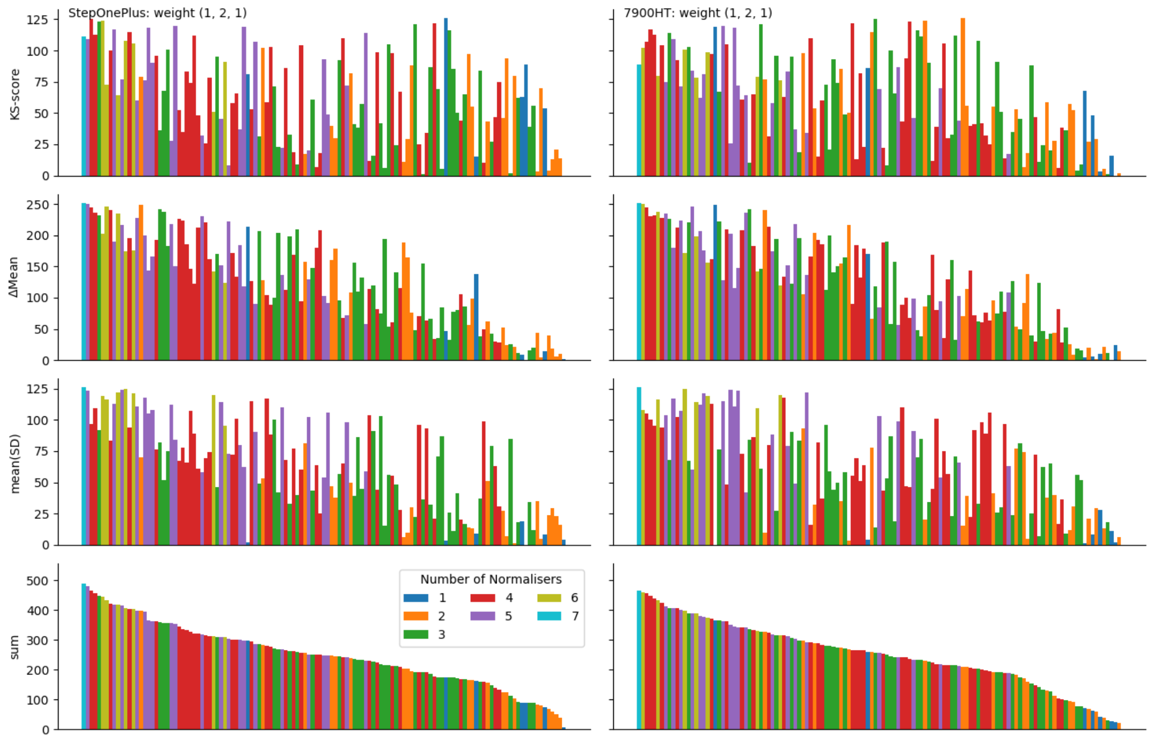

Supplementary Figure 5D. Individual and sum score components with weights 1; 2; 1.

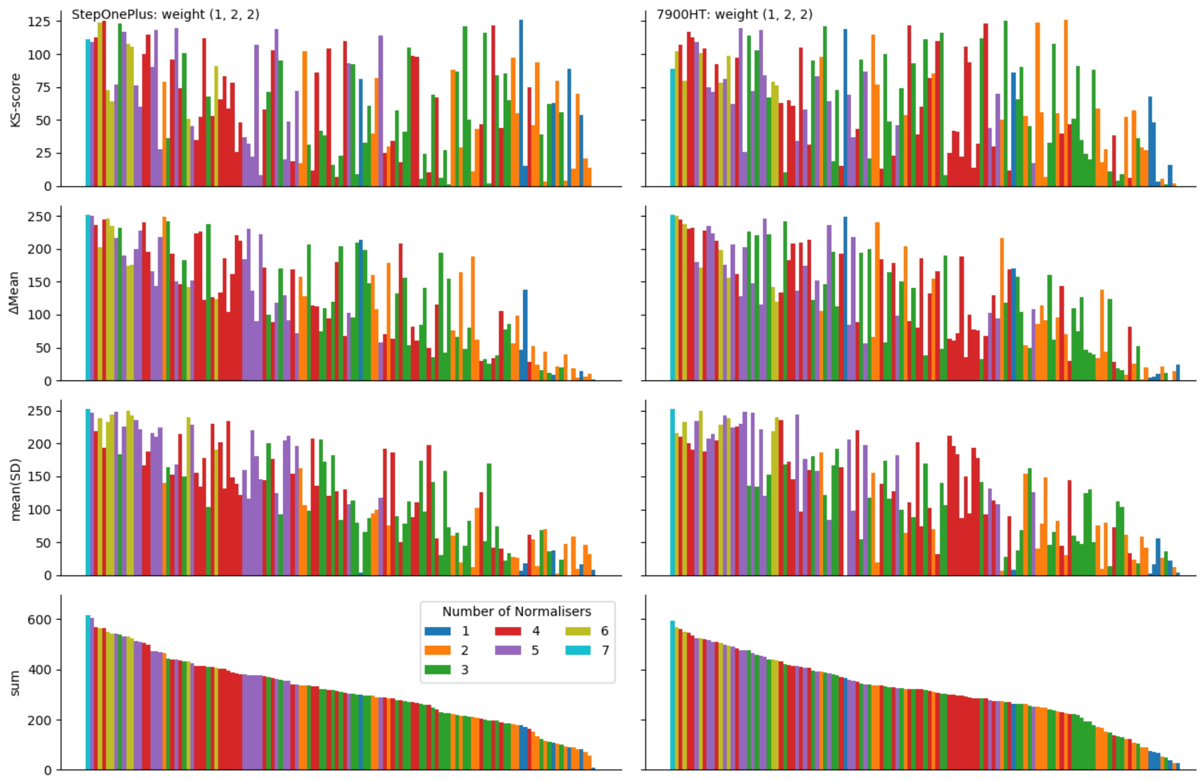

Supplementary Figure 5E. Individual and sum score components with weights 1; 2; 2.

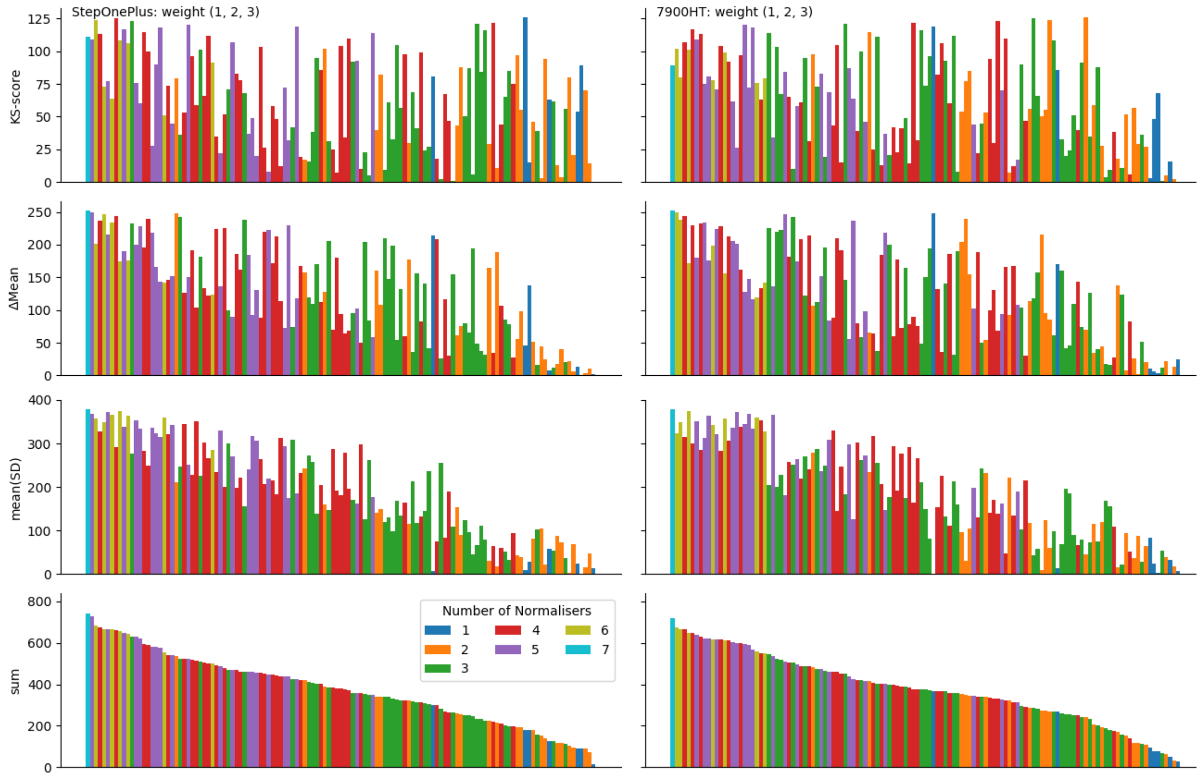

Supplementary Figure 5F. Individual and sum score components with weights 1; 2; 3.

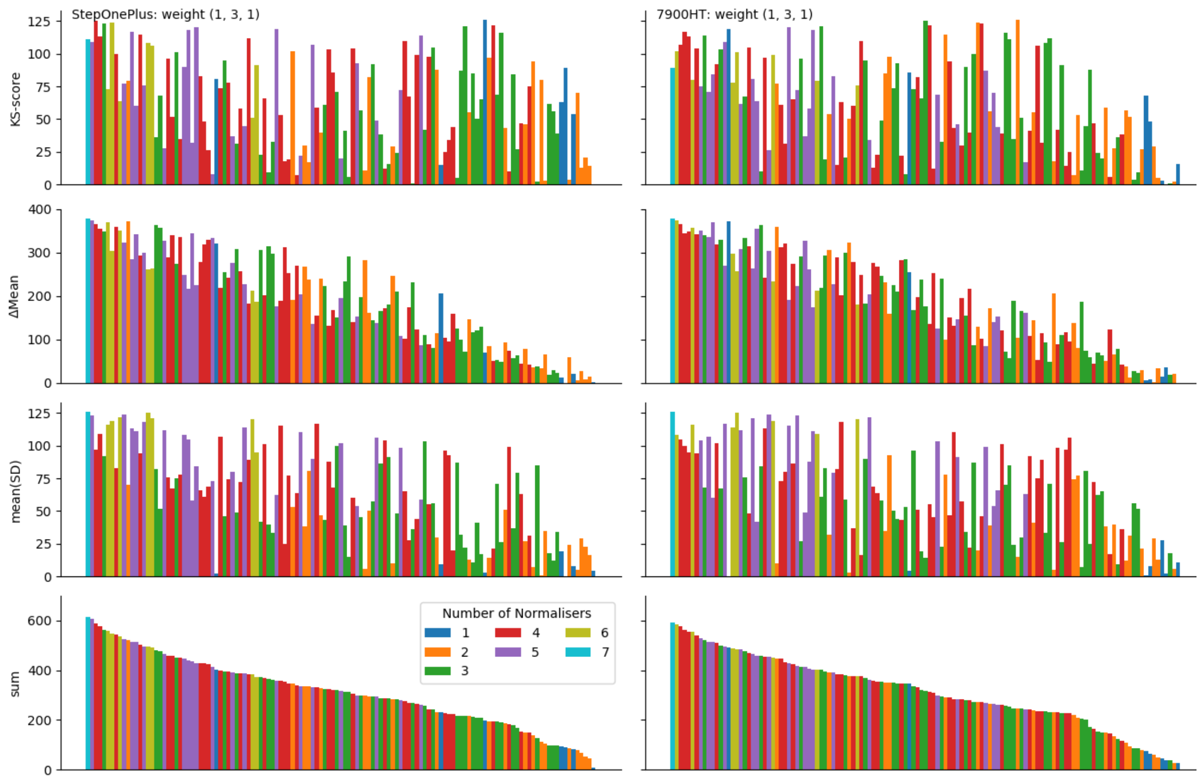

Supplementary Figure 5G. Individual and sum score components with weights 1; 3; 1.

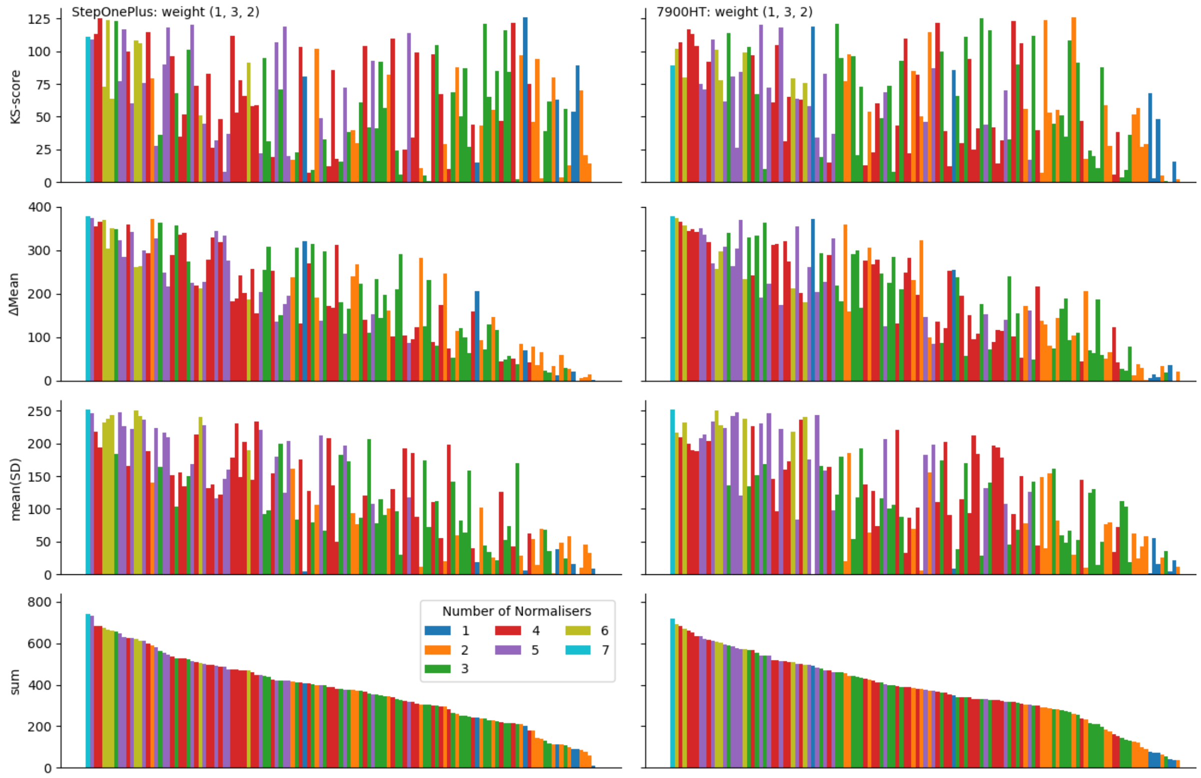

Supplementary Figure 5H. Individual and sum score components with weights 1; 3; 2.

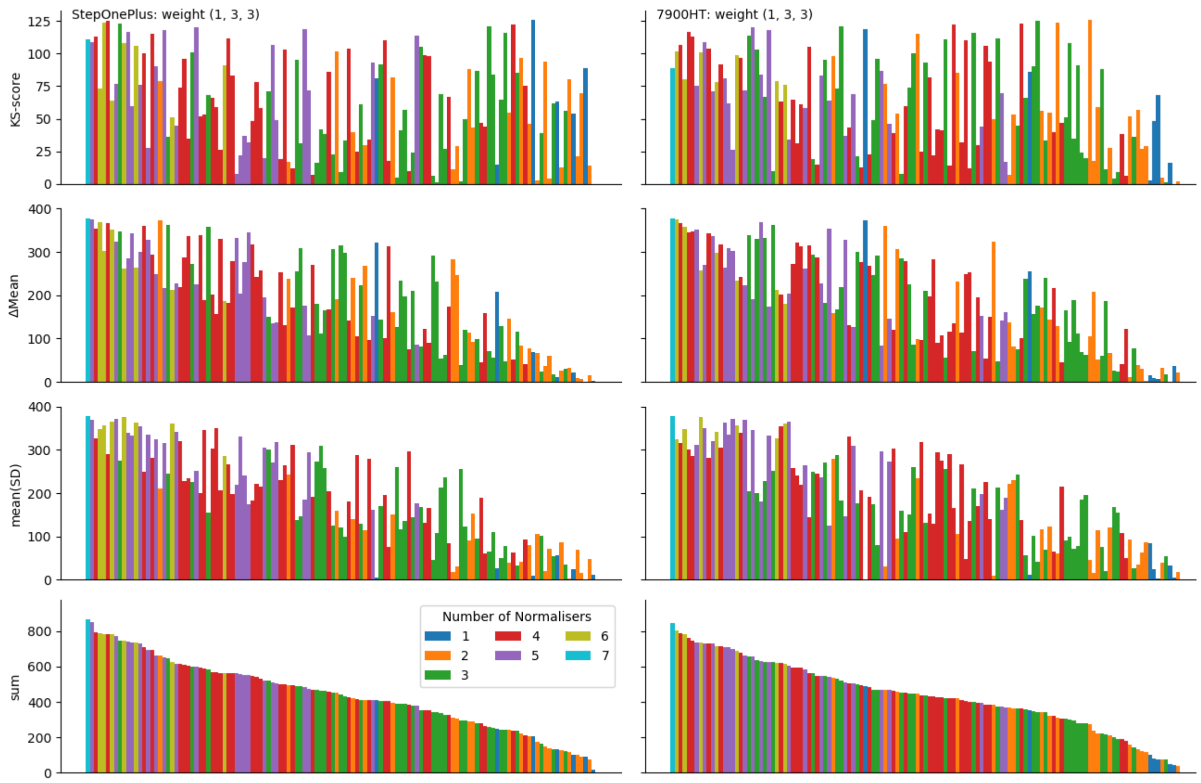

Supplementary Figure 5I. Individual and sum score components with weights 1; 3; 3.

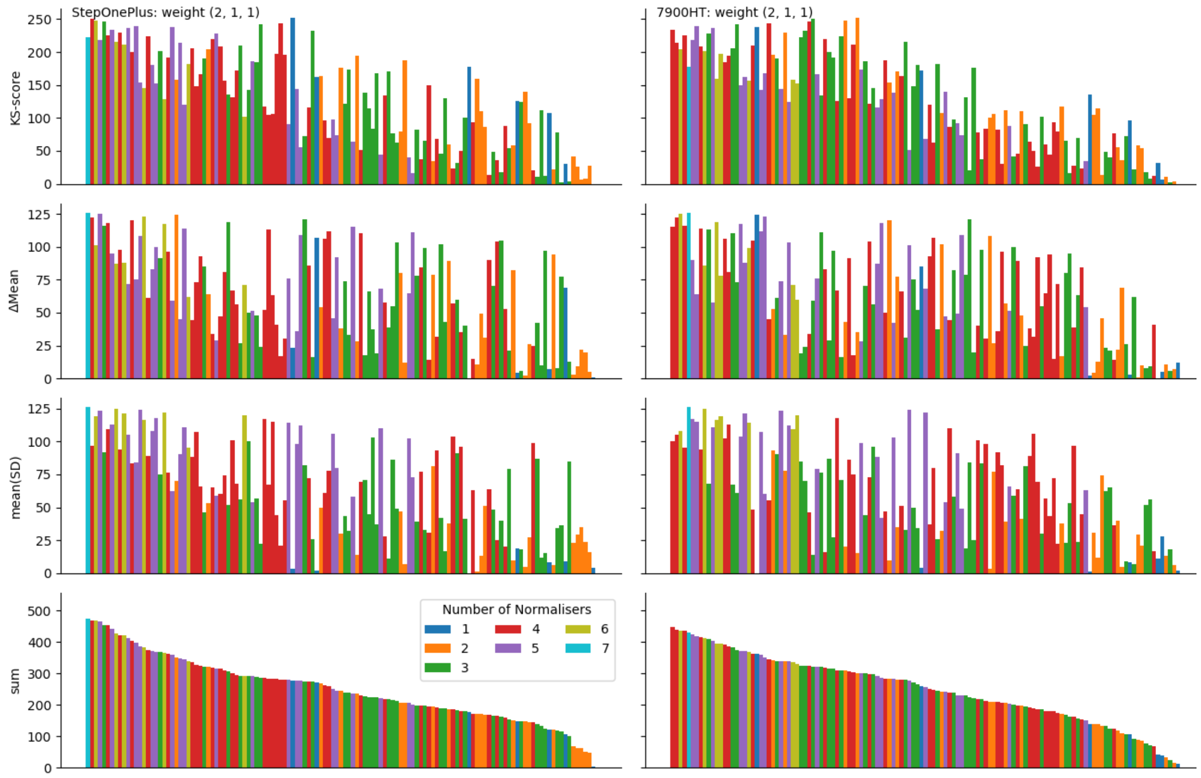

Supplementary Figure 5J. Individual and sum score components with weights 2; 1; 1.

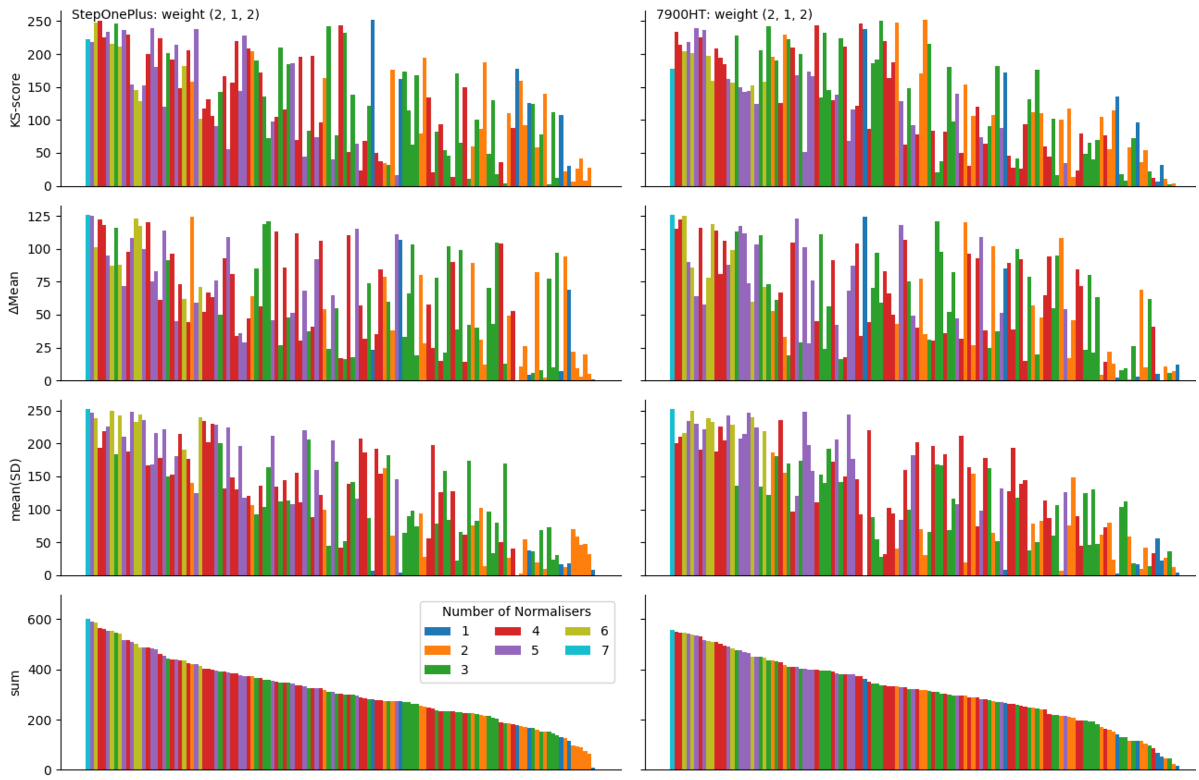

Supplementary Figure 5K. Individual and sum score components with weights 2; 1; 2.

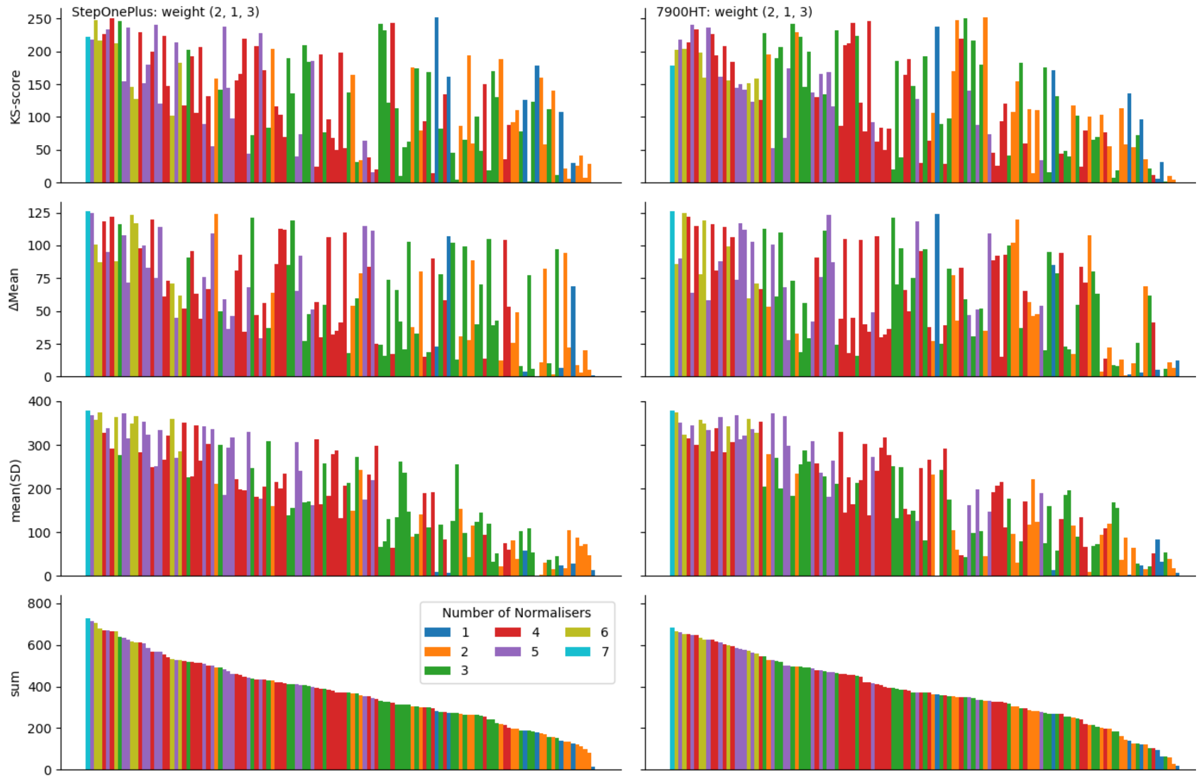

Supplementary Figure 5L. Individual and sum score components with weights 2; 1; 3.

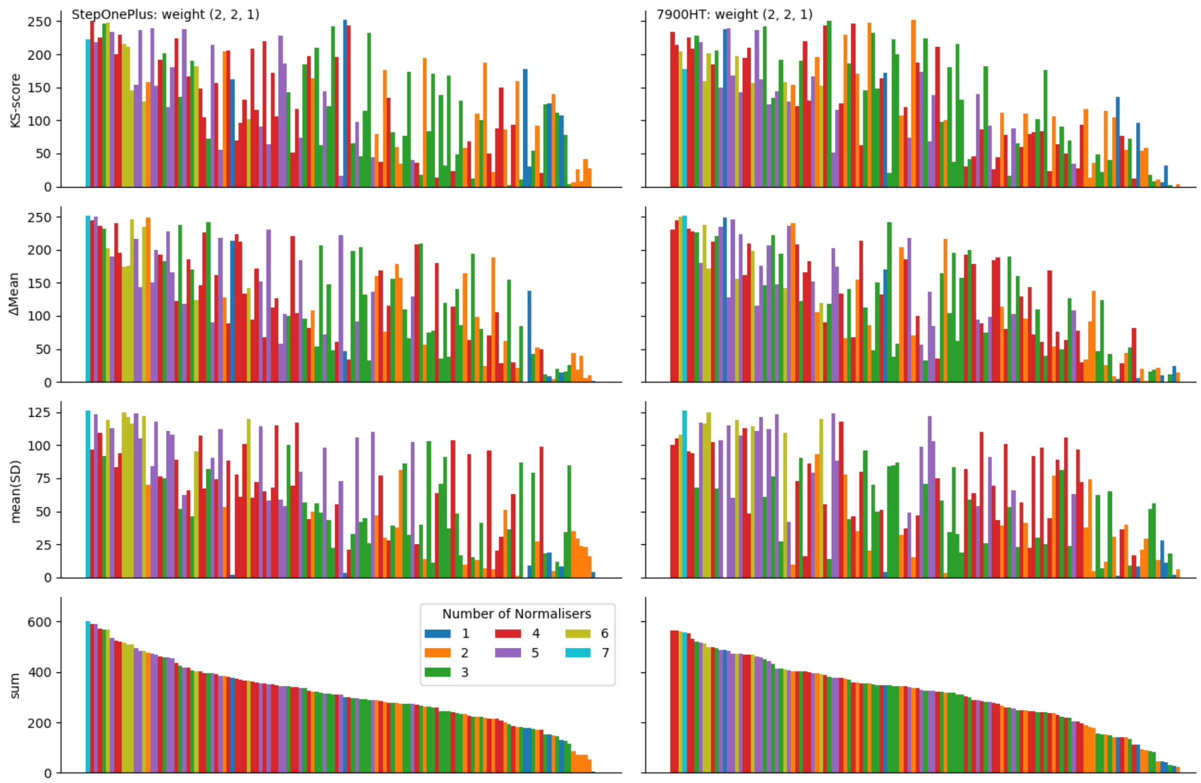

Supplementary Figure 5M. Individual and sum score components with weights 2; 2; 1.

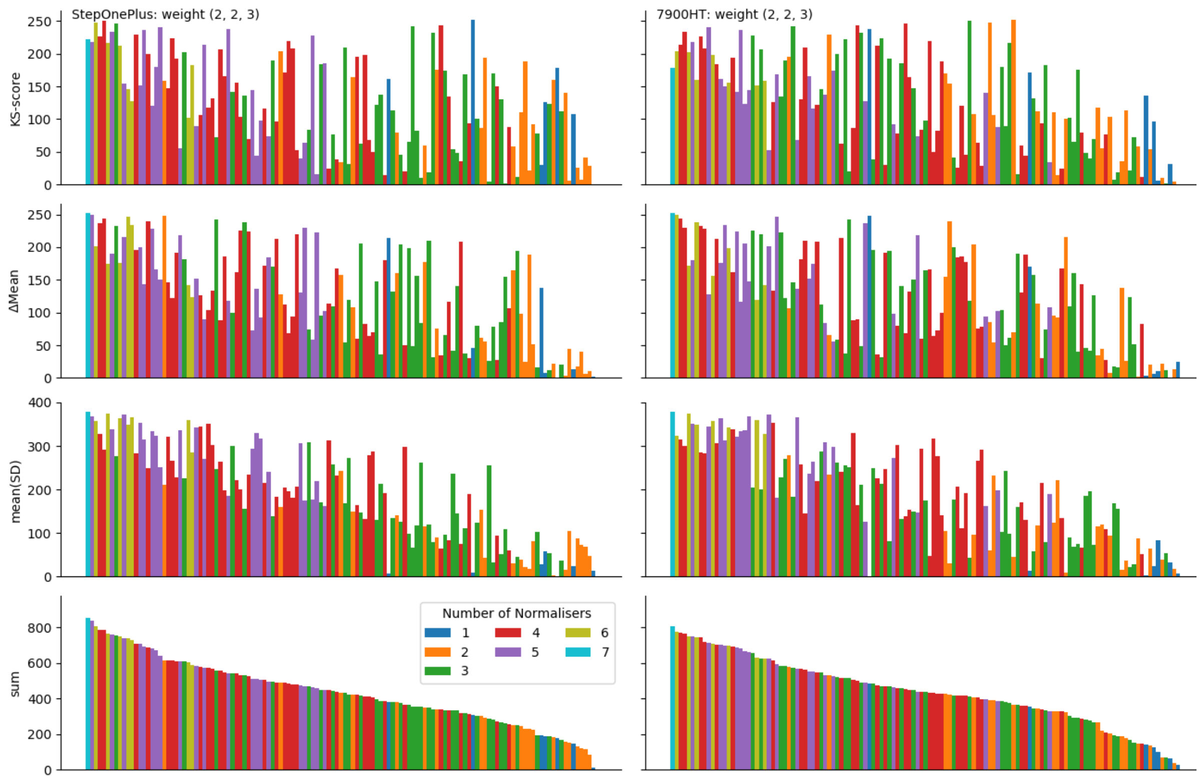

Supplementary Figure 5N. Individual and sum score components with weights 2; 2; 3.

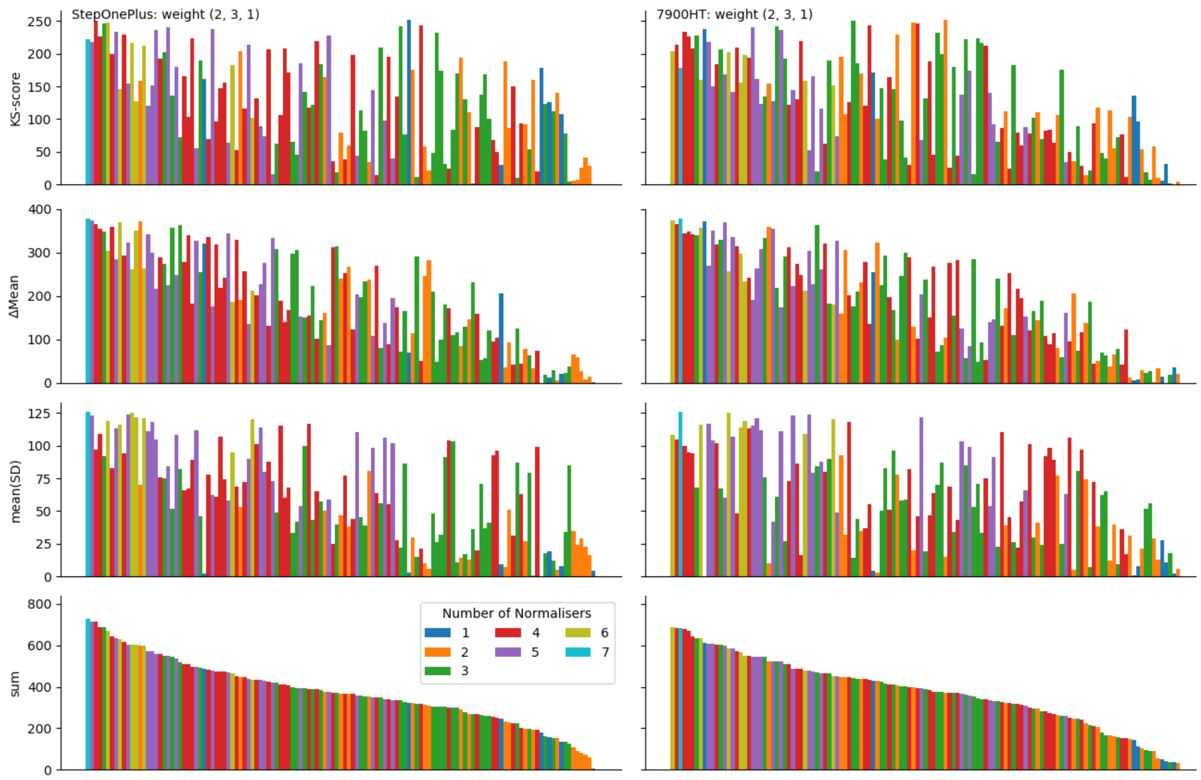

Supplementary Figure 5O. Individual and sum score components with weights 2; 3; 1.

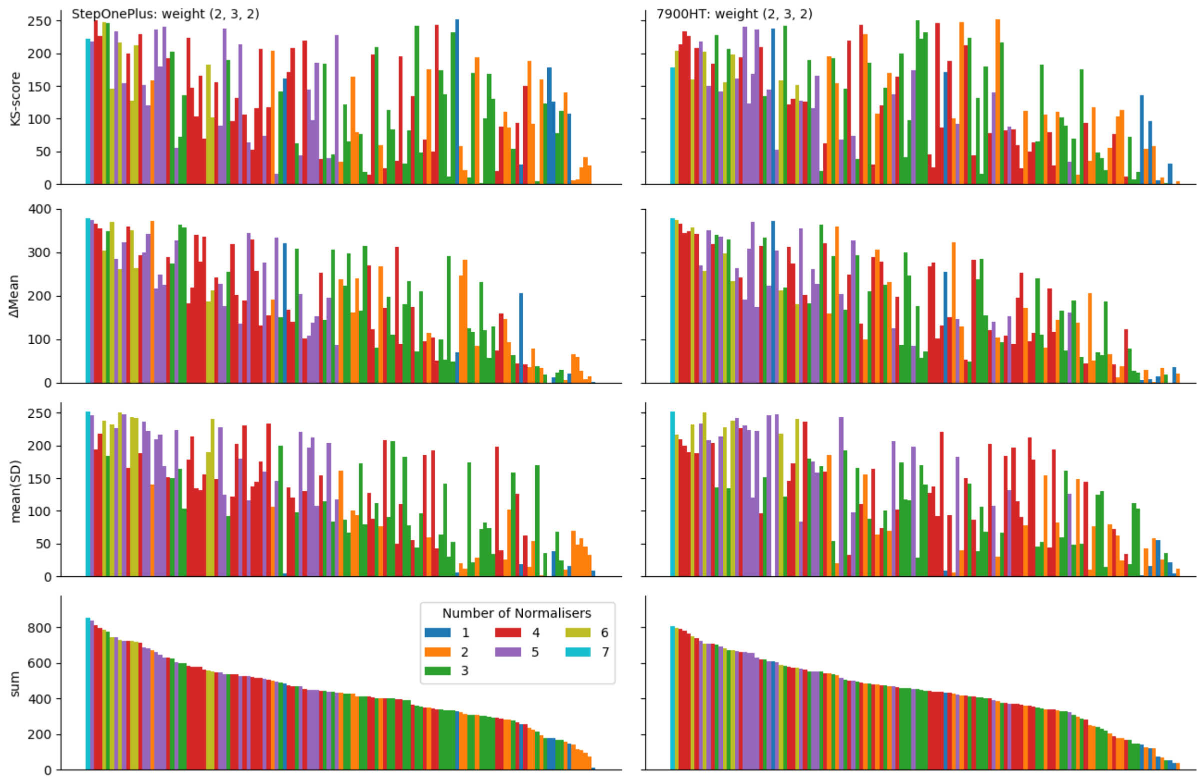

Supplementary Figure 5P. Individual and sum score components with weights 2; 3; 2.

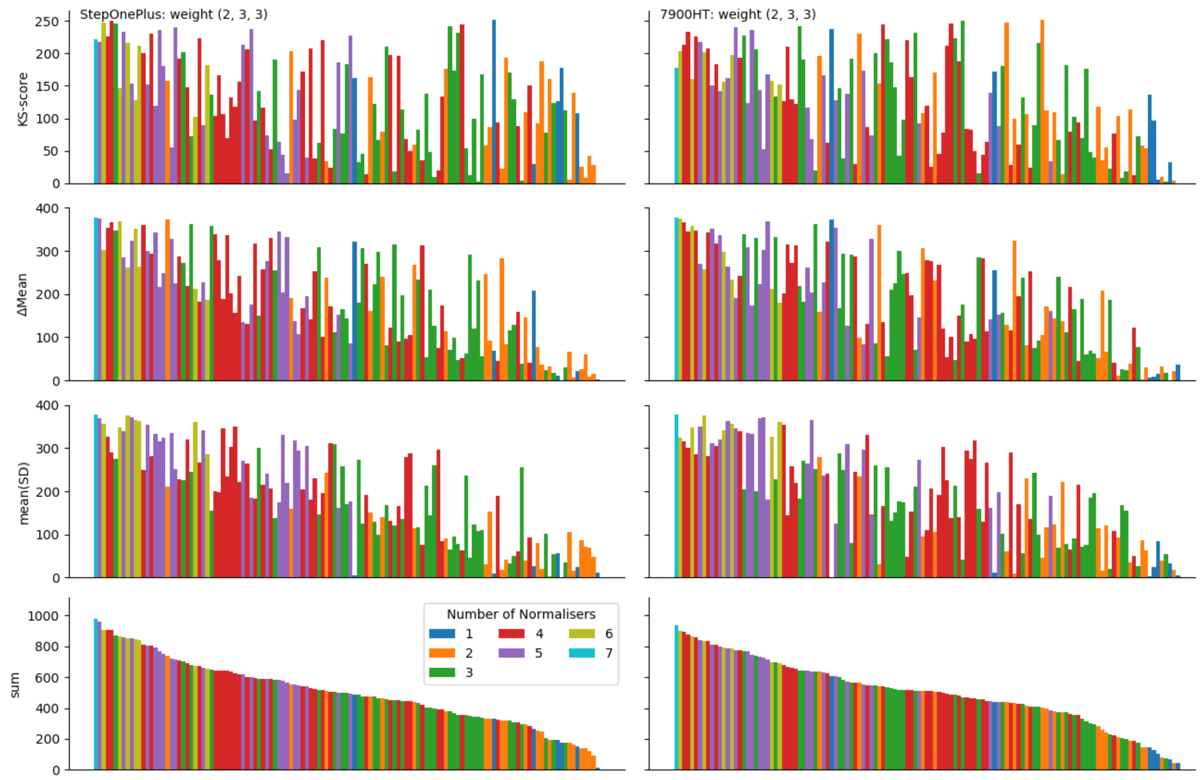

Supplementary Figure 5Q. Individual and sum score components with weights 2; 3; 3.

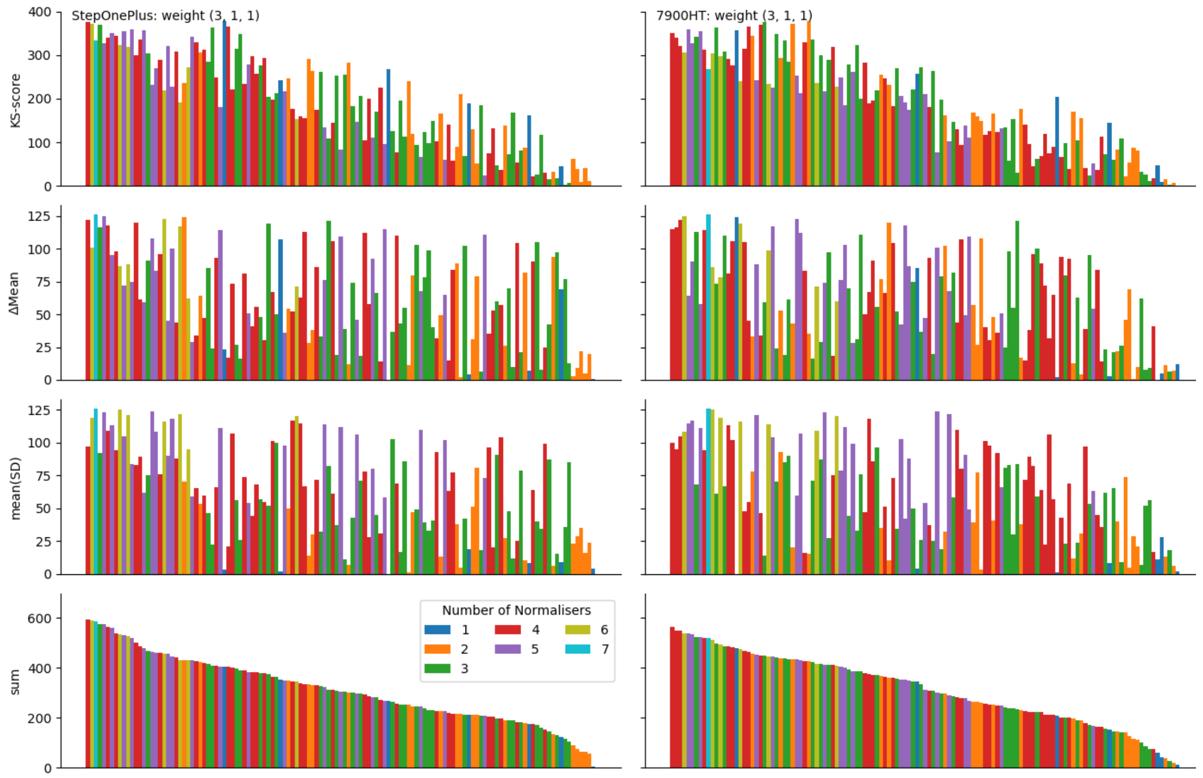

Supplementary Figure 5R. Individual and sum score components with weights 3; 1; 1.

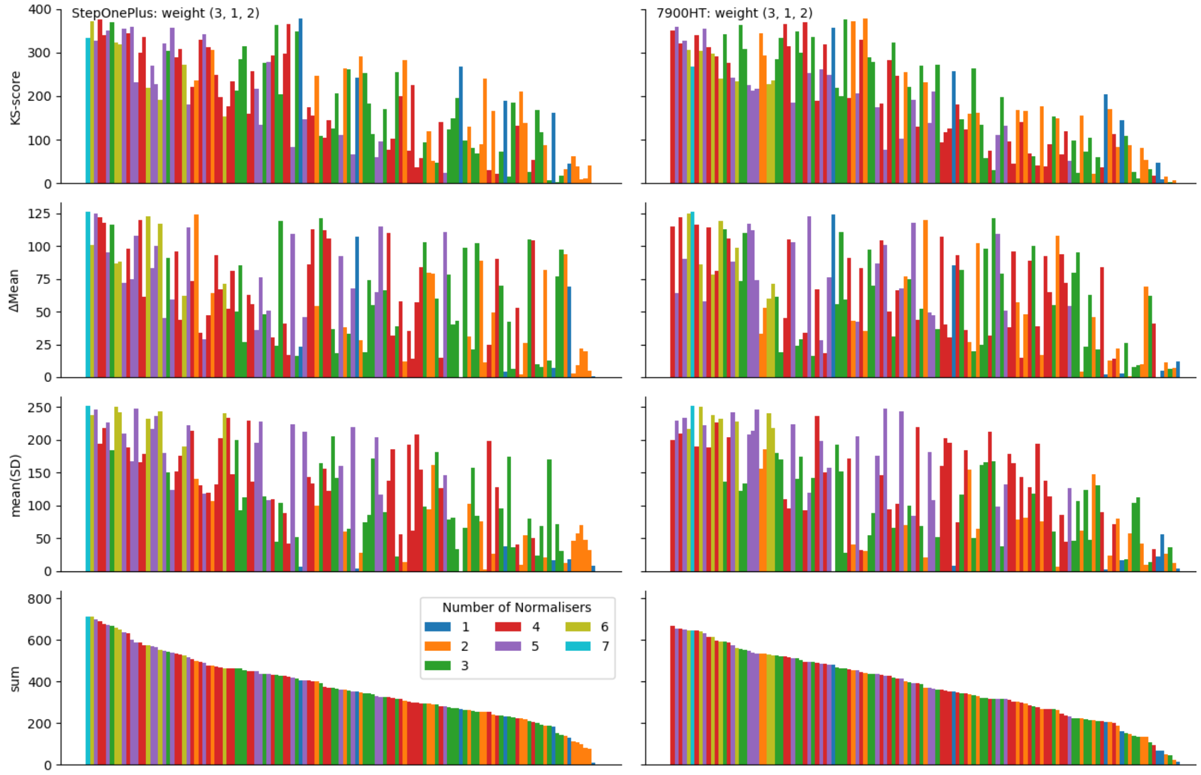

Supplementary Figure 5S. Individual and sum score components with weights 3; 1; 2.

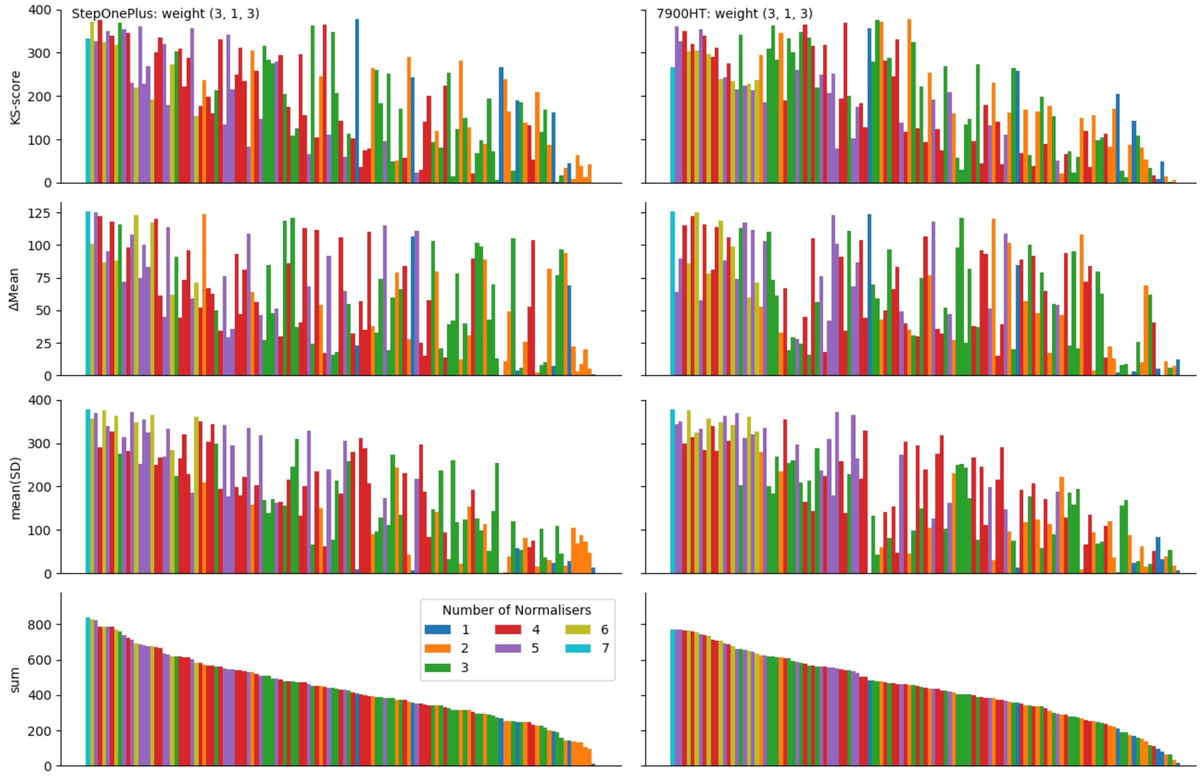

Supplementary Figure 5T. Individual and sum score components with weights 3; 1; 3.

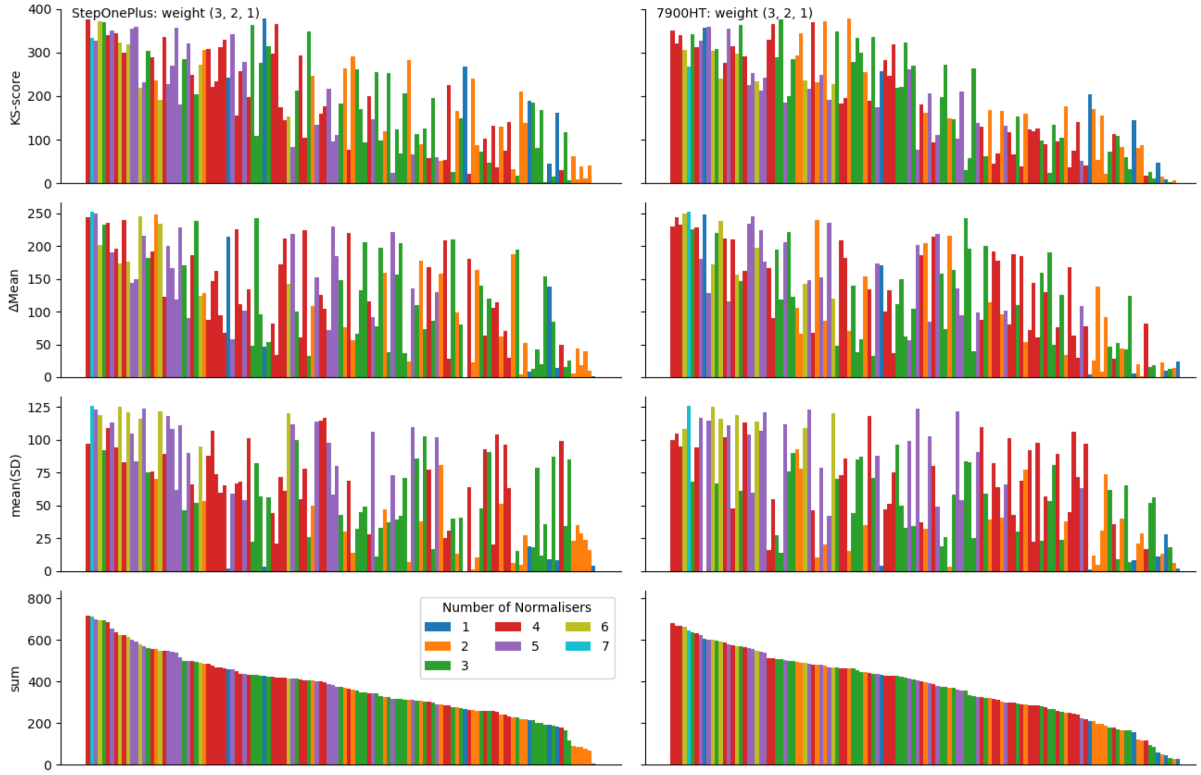

Supplementary Figure 5U. Individual and sum score components with weights 3; 2; 1.

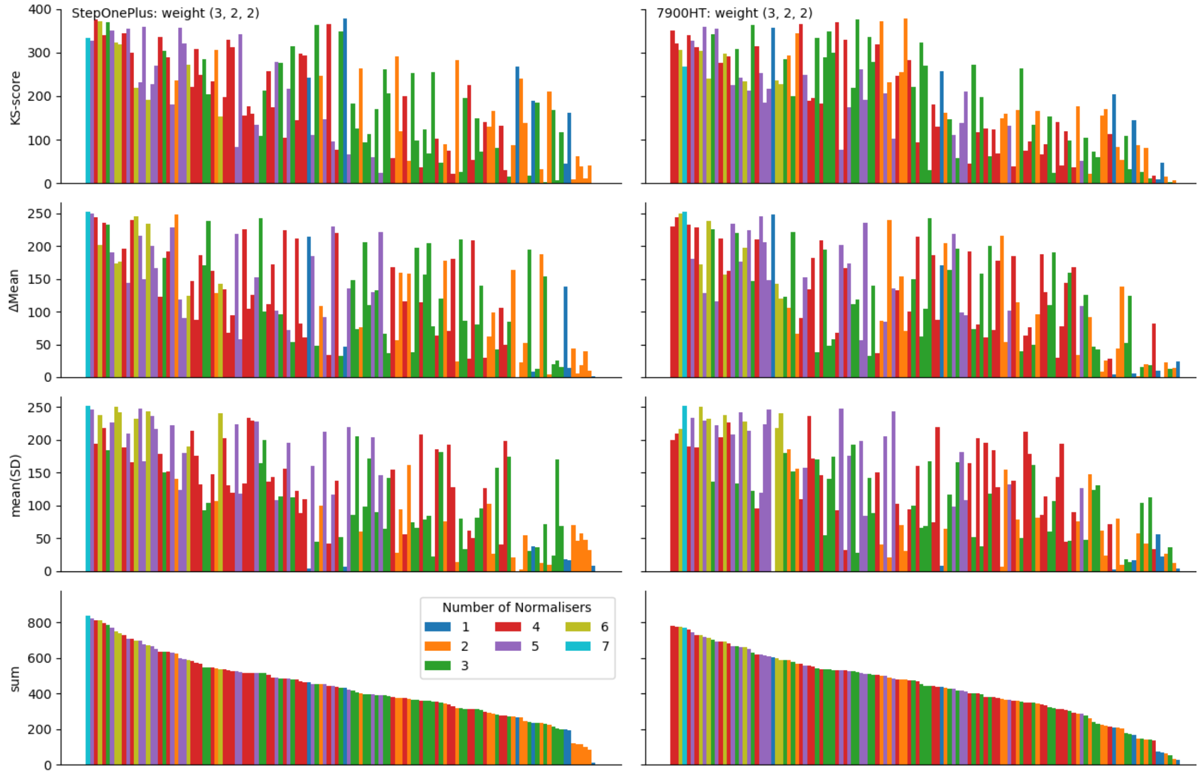

Supplementary Figure 5V. Individual and sum score components with weights 3; 2; 2.

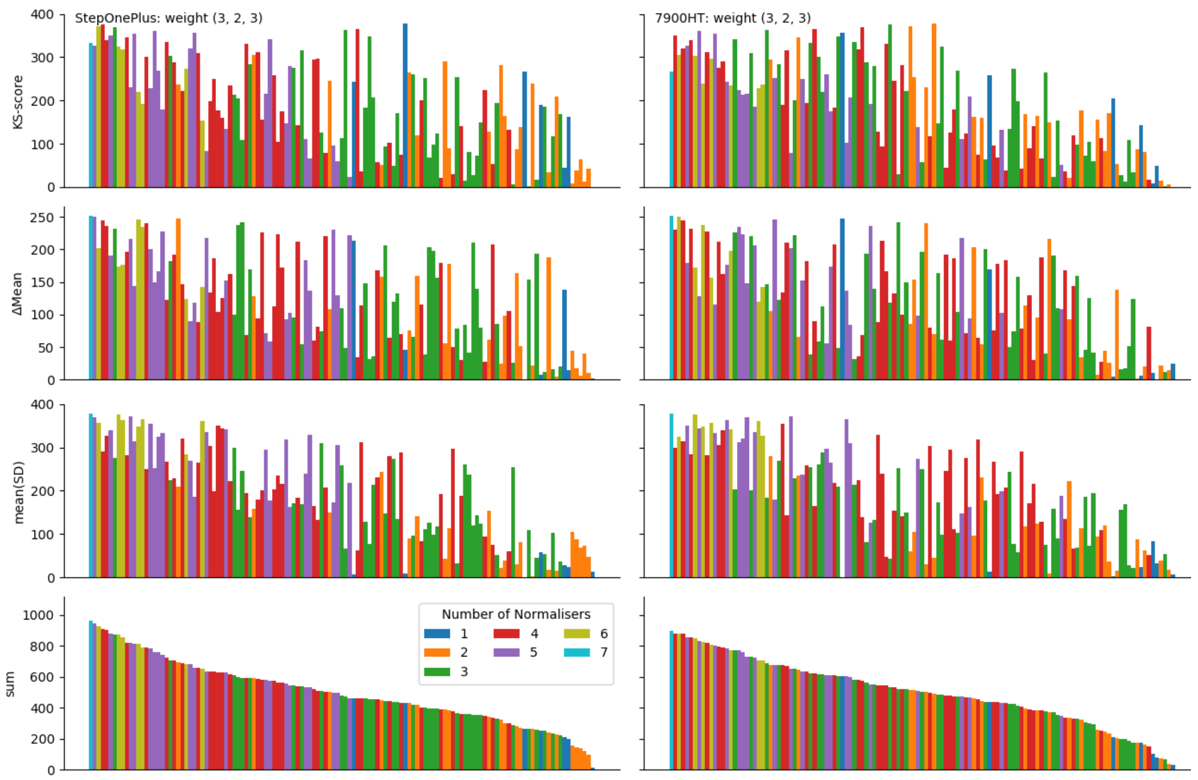

Supplementary Figure 5W. Individual and sum score components with weights 3; 2; 3.

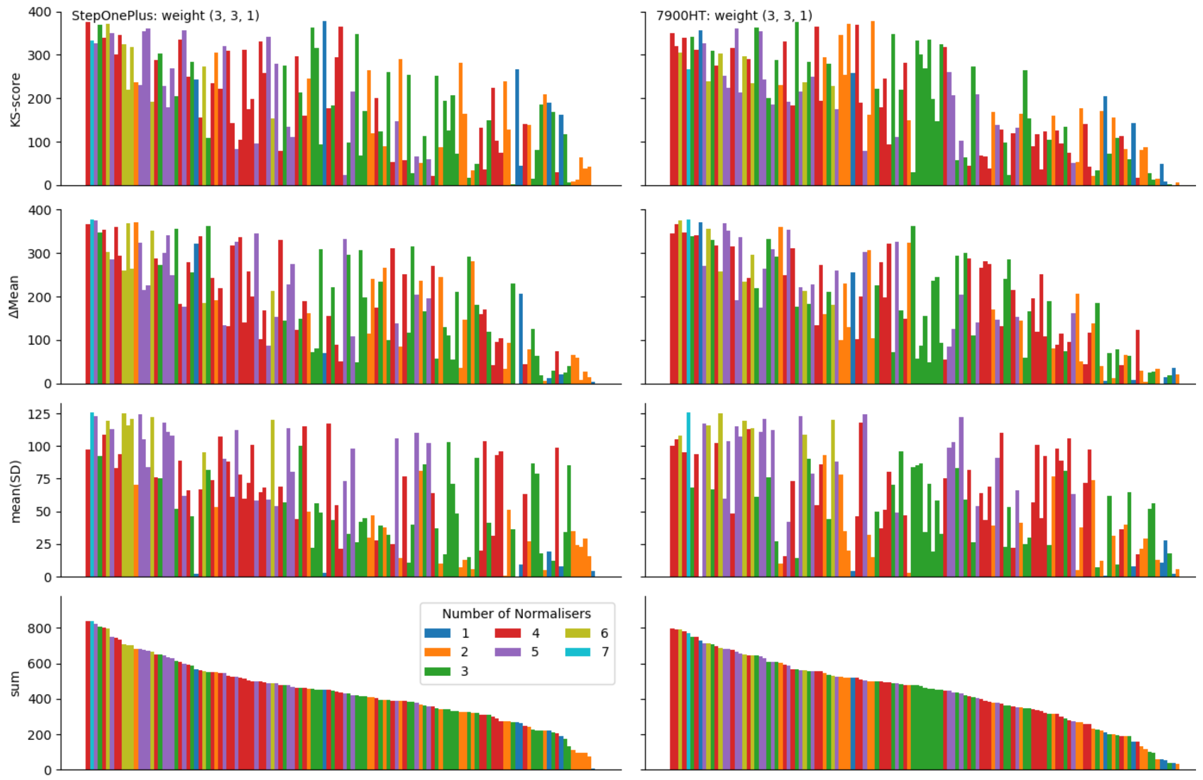

Supplementary Figure 5X. Individual and sum score components with weights 3; 3; 1.

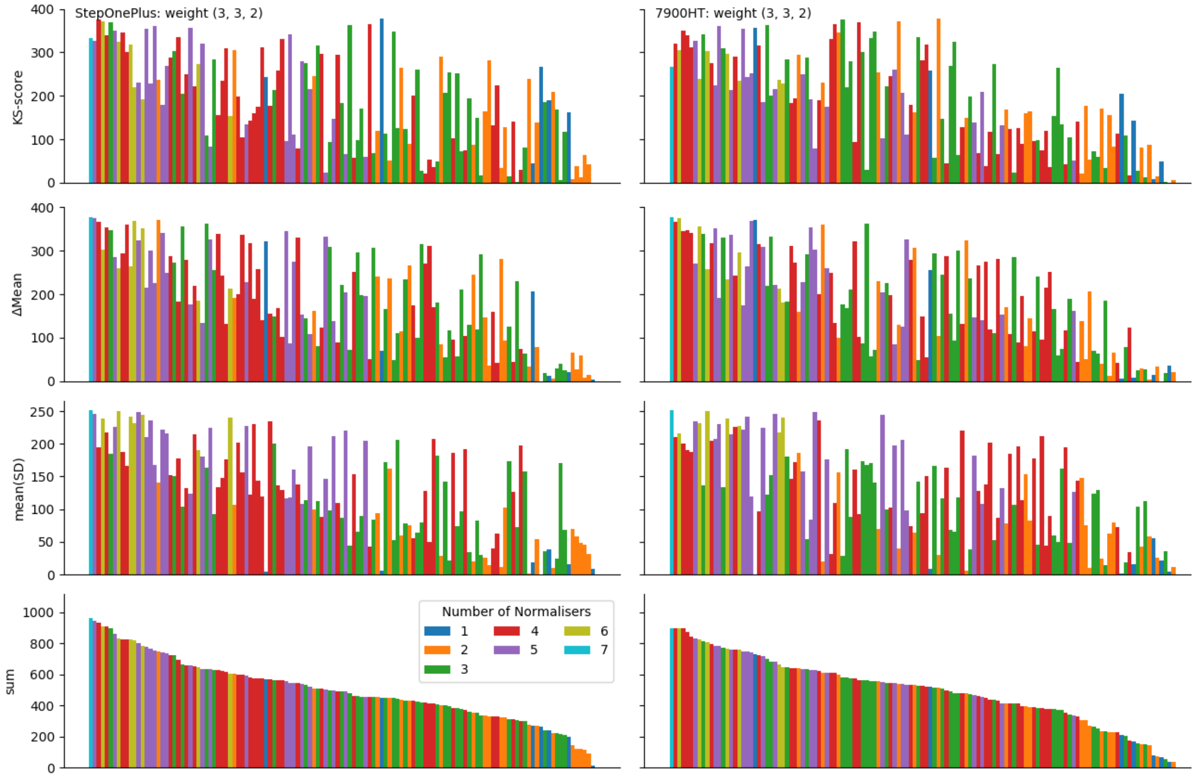

Supplementary Figure 5Y. Individual and sum score components with weights 3; 3; 2.

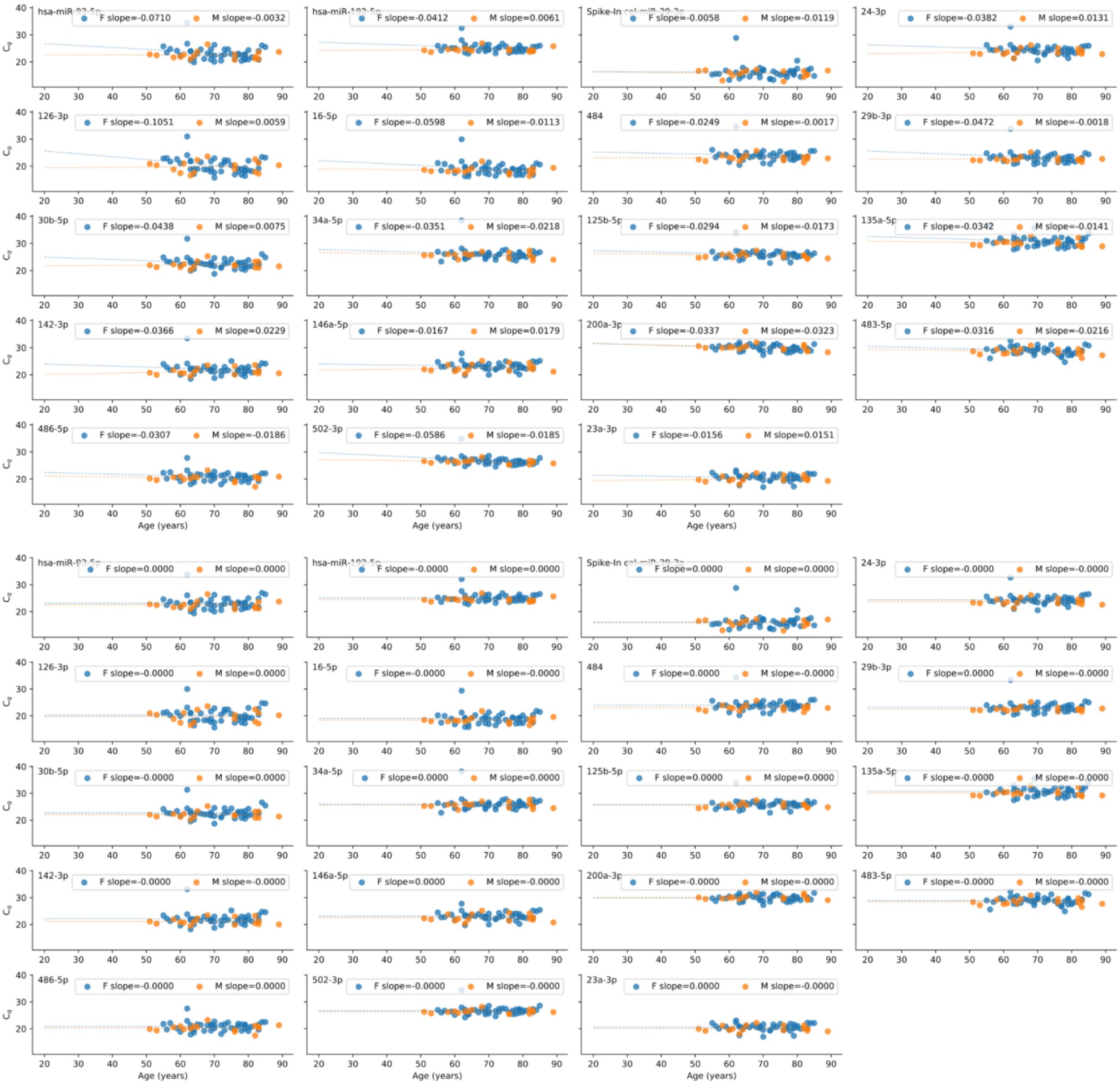

Supplementary Figure 6A - Białystok ExpressionSuite Cq Age adjustment (top: pre- and bottom: post-adjustment. Age adjustment was performed separately for Male and Female subjects.

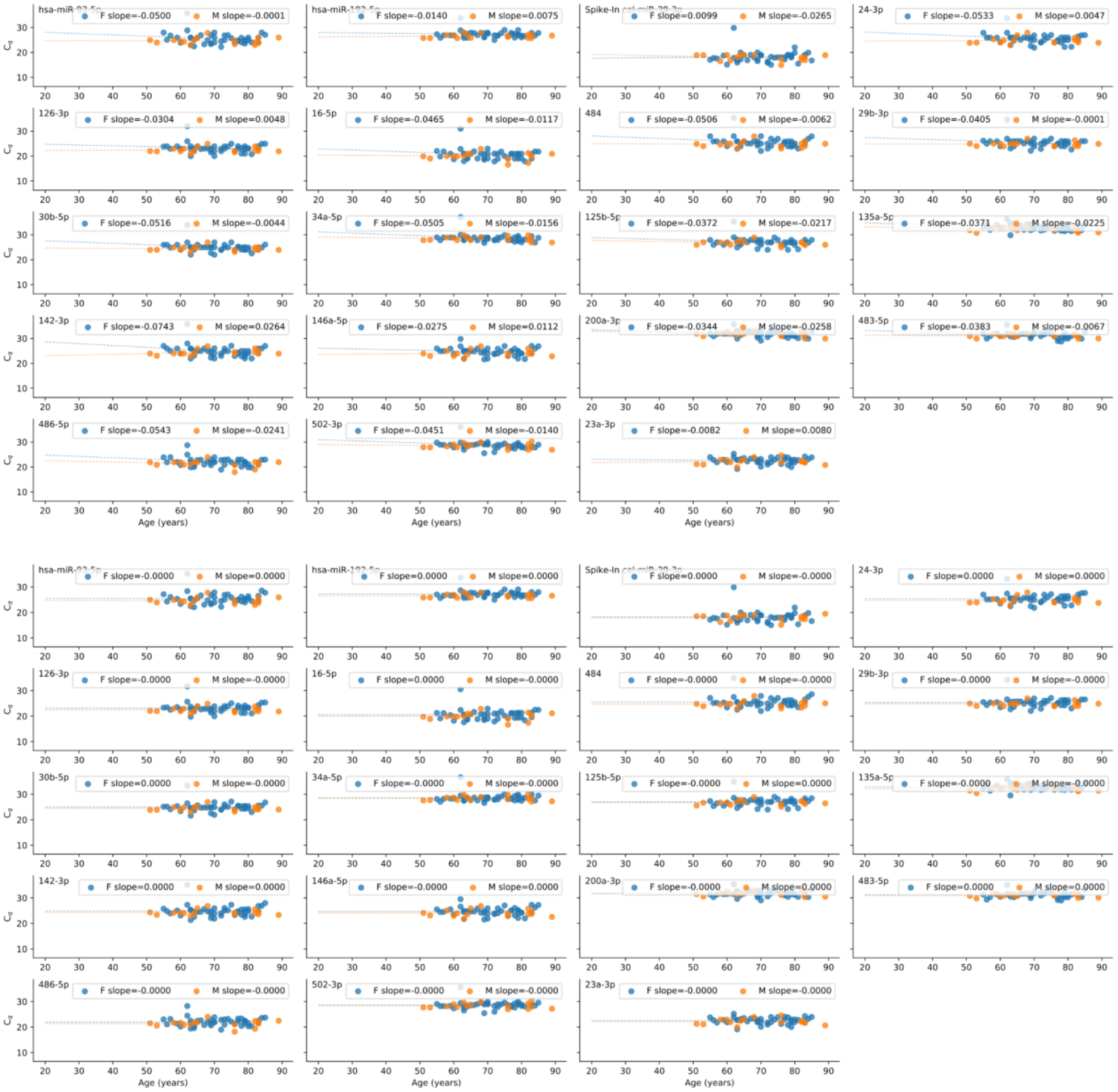

Supplementary Figure 6B - Białystok SDS Cq Age adjustment (top: pre- and bottom: post-adjustment. Age adjustment was performed separately for Male and Female subjects.

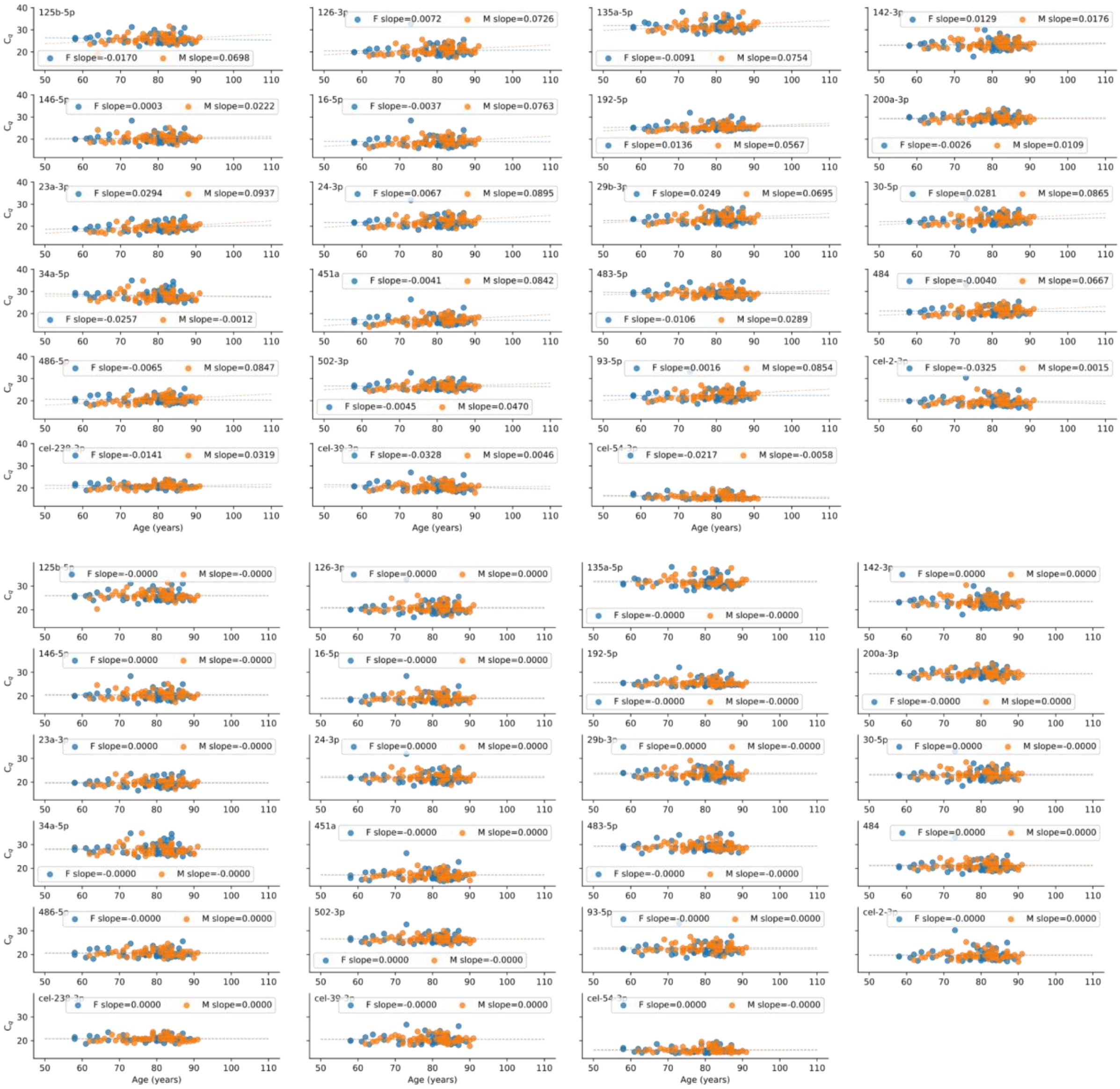

Supplementary Figure 6C - Warsaw Cohort ExpressionSuite Cq Age adjustment (top: pre- and bottom: post-adjustment. Age adjustment was performed separately for Male and Female subjects.

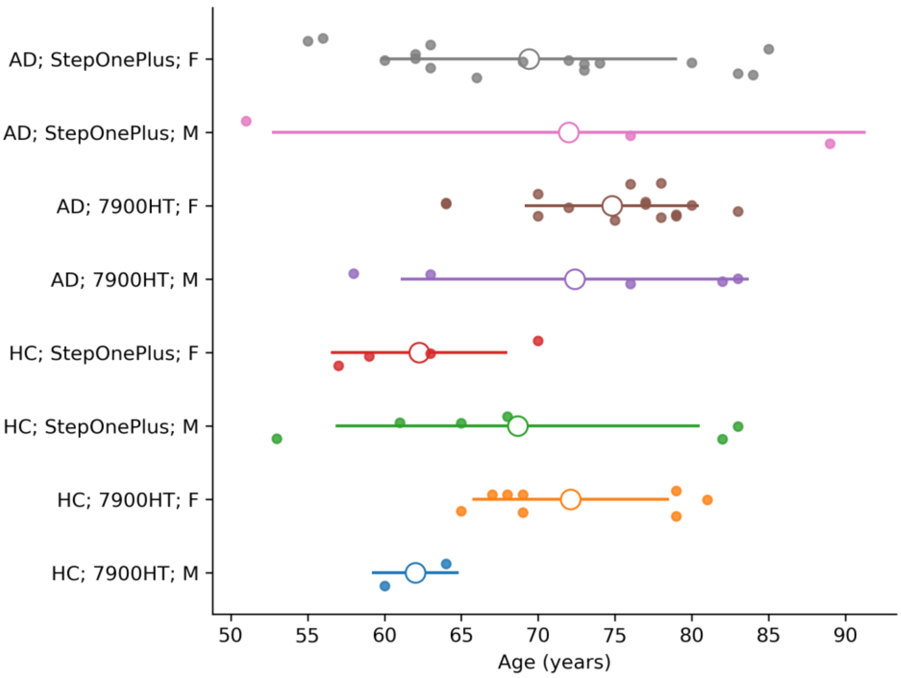


```
diagnosis ~ age * MMSE + (1|machine)

Random Effects
Groups      Names            Variance    Std.Dev
Machine     (Intercept)         0           0
Number of obs: 60, groups:  machine, 2
```

Fixed Effects	Estimate	Std.	Error	z-value	Pr(>|z|)
(Intercept)	-1.17E+02	1.33E+01	-8.841	< 0.0000000000000002	***
age	1.22E+00	1.53E-01	7.984	< 0.00000000000000141	***
MMSE	4.70E+00	5.18E-01	9.07	< 0.0000000000000002	***
age:MMSE	-4.98E-02	5.57E-03	-8.936	< 0.0000000000000002	***

Signif. Codes '***' 0.001 '**' 0.01 '*' 0.05 '.' 0.1

Supplementary Figure 7. Analysis of subject age separated by analysis machine, and by all subgroup delineations (disease, machine, sex) supported by creation of a generalised linear mixed model in R (4.0.5) using the lme4 package, specified as detailed below the plot.

Supplementary Table 1. Summary statistics for Haemolysis measures shown in Figure 1B

	Machine	*Values*		
	ExSuite		SDS	
Diagnosis	Mean	CV	Mean	CV
AD	4.797758035	31.90027342	6.819101266	35.92185398
HC	4.644301583	28.28119432	6.629989886	27.35640156

Supplementary Table 2. Summary statistics for data shown in Figure 1C-E

	Software	*Values*		
	ExSuite		SDS	
Normaliser	Mean	CV	Mean	CV
484	23.59223599	5.397717046	25.18691263	5.092193072
126-3p	19.97226389	10.86857094	22.79581258	4.905092457
16-5p	18.81976108	7.347544544	20.52760887	5.381771019
23a-3p	20.33155591	5.812925427	22.39922257	4.944258296
24-3p	24.00648412	5.302180258	25.1624251	5.28919145
hsa-miR-192-5p	24.94688436	4.042463989	27.19982407	2.600736573
hsa-miR-93-5p	22.91630089	7.413378556	25.17692474	5.700693427

Supplementary Table 3. Resource for translation of numerical aliases to combinations of normalisers, for 7-normaliser comparison.

numerical code	93-5p	192-5p	24-3p	126-3p	16-5p	484	23a-3p
0	93-5p						
1		192-5p					
2			24-3p				
3				126-3p			
4					16-5p		
5						484	

6							23a-3p
7	93-5p	192-5p					
8	93-5p		24-3p				
9	93-5p			126-3p			
10	93-5p				16-5p		
11	93-5p					484	
12	93-5p						23a-3p
13		192-5p	24-3p				
14		192-5p		126-3p			
15		192-5p			16-5p		
16		192-5p				484	
17		192-5p					23a-3p
18			24-3p	126-3p			
19			24-3p		16-5p		
20			24-3p			484	
21			24-3p				23a-3p
22				126-3p	16-5p		
numerical code	93-5p	192-5p	24-3p	126-3p	16-5p	484	23a-3p
23				126-3p		484	
24				126-3p			23a-3p

25					16-5p	484	
26					16-5p		23a-3p
27						484	23a-3p
28	93-5p	192-5p	24-3p				
29	93-5p	192-5p		126-3p			
30	93-5p	192-5p			16-5p		
31	93-5p	192-5p				484	
32	93-5p	192-5p					23a-3p
33	93-5p		24-3p	126-3p			
34	93-5p		24-3p		16-5p		
35	93-5p		24-3p			484	
36	93-5p		24-3p				23a-3p
37	93-5p			126-3p	16-5p		
38	93-5p			126-3p		484	
39	93-5p			126-3p			23a-3p
40	93-5p				16-5p	484	
41	93-5p				16-5p		23a-3p
42	93-5p					484	23a-3p
43		192-5p	24-3p	126-3p			
44		192-5p	24-3p		16-5p		

45		192-5p	24-3p			484	
46		192-5p	24-3p				23a-3p
47		192-5p		126-3p	16-5p		
48		192-5p		126-3p		484	
numerical code	93-5p	192-5p	24-3p	126-3p	16-5p	484	23a-3p
49		192-5p		126-3p			23a-3p
50		192-5p			16-5p	484	
51		192-5p			16-5p		23a-3p
52		192-5p				484	23a-3p
53			24-3p	126-3p	16-5p		
54			24-3p	126-3p		484	
55			24-3p	126-3p			23a-3p
56			24-3p		16-5p	484	
57			24-3p		16-5p		23a-3p
58			24-3p			484	23a-3p
59				126-3p	16-5p	484	
60				126-3p	16-5p		23a-3p
61				126-3p		484	23a-3p
62					16-5p	484	23a-3p
63	93-5p	192-5p	24-3p	126-3p			

64	93-5p	192-5p	24-3p		16-5p		
65	93-5p	192-5p	24-3p			484	
66	93-5p	192-5p	24-3p				23a-3p
67	93-5p	192-5p		126-3p	16-5p		
68	93-5p	192-5p		126-3p		484	
69	93-5p	192-5p		126-3p			23a-3p
70	93-5p	192-5p			16-5p	484	
71	93-5p	192-5p			16-5p		23a-3p
72	93-5p	192-5p				484	23a-3p
73	93-5p		24-3p	126-3p	16-5p		
74	93-5p		24-3p	126-3p		484	
numerical code	93-5p	192-5p	24-3p	126-3p	16-5p	484	23a-3p
75	93-5p		24-3p	126-3p			23a-3p
76	93-5p		24-3p		16-5p	484	
77	93-5p		24-3p		16-5p		23a-3p
78	93-5p		24-3p			484	23a-3p
79	93-5p			126-3p	16-5p	484	
80	93-5p			126-3p	16-5p		23a-3p
81	93-5p			126-3p		484	23a-3p
82	93-5p				16-5p	484	23a-3p

83		192-5p	24-3p	126-3p	16-5p		
84		192-5p	24-3p	126-3p		484	
85		192-5p	24-3p	126-3p			23a-3p
86		192-5p	24-3p		16-5p	484	
87		192-5p	24-3p		16-5p		23a-3p
88		192-5p	24-3p			484	23a-3p
89		192-5p		126-3p	16-5p	484	
90		192-5p		126-3p	16-5p		23a-3p
91		192-5p		126-3p		484	23a-3p
92		192-5p			16-5p	484	23a-3p
93			24-3p	126-3p	16-5p	484	
94			24-3p	126-3p	16-5p		23a-3p
95			24-3p	126-3p		484	23a-3p
96			24-3p		16-5p	484	23a-3p
97				126-3p	16-5p	484	23a-3p
98	93-5p	192-5p	24-3p	126-3p	16-5p		
99	93-5p	192-5p	24-3p	126-3p		484	
100	93-5p	192-5p	24-3p	126-3p			23a-3p
numerical code	93-5p	192-5p	24-3p	126-3p	16-5p	484	23a-3p
101	93-5p	192-5p	24-3p		16-5p	484	

102	93-5p	192-5p	24-3p		16-5p		23a-3p
103	93-5p	192-5p	24-3p			484	23a-3p
104	93-5p	192-5p		126-3p	16-5p	484	
105	93-5p	192-5p		126-3p	16-5p		23a-3p
106	93-5p	192-5p		126-3p		484	23a-3p
107	93-5p	192-5p			16-5p	484	23a-3p
108	93-5p		24-3p	126-3p	16-5p	484	
109	93-5p		24-3p	126-3p	16-5p		23a-3p
110	93-5p		24-3p	126-3p		484	23a-3p
111	93-5p		24-3p		16-5p	484	23a-3p
112	93-5p			126-3p	16-5p	484	23a-3p
113		192-5p	24-3p	126-3p	16-5p	484	
114		192-5p	24-3p	126-3p	16-5p		23a-3p
115		192-5p	24-3p	126-3p		484	23a-3p
116		192-5p	24-3p		16-5p	484	23a-3p
117		192-5p		126-3p	16-5p	484	23a-3p
118			24-3p	126-3p	16-5p	484	23a-3p
119	93-5p	192-5p	24-3p	126-3p	16-5p	484	
120	93-5p	192-5p	24-3p	126-3p	16-5p		23a-3p
121	93-5p	192-5p	24-3p	126-3p		484	23a-3p

122	93-5p	192-5p	24-3p		16-5p	484	23a-3p
123	93-5p	192-5p		126-3p	16-5p	484	23a-3p
124	93-5p		24-3p	126-3p	16-5p	484	23a-3p
125		192-5p	24-3p	126-3p	16-5p	484	23a-3p
126	93-5p	192-5p	24-3p	126-3p	16-5p	484	23a-3p
